# Bright Light Increases Alertness and Not Cortisol in Healthy Men: A Forced Desynchrony Study Under Dim and Bright Light (I)

**DOI:** 10.1177/07487304221096945

**Published:** 2022-06-10

**Authors:** R. Lok, T. Woelders, M. J. van Koningsveld, K. Oberman, S. G. Fuhler, D. G. M. Beersma, R. A. Hut

**Affiliations:** * Chronobiology Unit, Groningen Institute for Evolutionary Life Sciences, University of Groningen, Groningen, the Netherlands; † Current address: Department of Psychiatry and Behavioral Sciences, Stanford University, Palo Alto, California, USA; ‡ University of Groningen, Leeuwarden, the Netherlands

**Keywords:** light, wake duration related variation, circadian variation, forced desynchrony, subjective alertness, performance

## Abstract

Light-induced improvements in alertness are more prominent during nighttime than during the day, suggesting that alerting effects of light may depend on internal clock time or wake duration. Relative contributions of both factors can be quantified using a forced desynchrony (FD) designs. FD designs have only been conducted under dim light conditions (<10 lux) since light above this amount can induce non-uniform phase progression of the circadian pacemaker (also called relative coordination). This complicates the mathematical separation of circadian clock phase from homeostatic sleep pressure effects. Here we investigate alerting effects of light in a novel 4 × 18 h FD protocol (5 h sleep, 13 h wake) under dim (6 lux) and bright light (1300 lux) conditions. Hourly saliva samples (melatonin and cortisol assessment) and 2-hourly test sessions were used to assess effects of bright light on subjective and objective alertness (electroencephalography and performance). Results reveal (1) stable free-running cortisol rhythms with uniform phase progression under both light conditions, suggesting that FD designs can be conducted under bright light conditions (1300 lux), (2) subjective alerting effects of light depend on elapsed time awake but not circadian clock phase, while (3) light consistently improves objective alertness independent of time awake or circadian clock phase. Reconstructing the daily time course by combining circadian clock phase and wake duration effects indicates that performance is improved during daytime, while subjective alertness remains unchanged. This suggests that high-intensity indoor lighting during the regular day might be beneficial for mental performance, even though this may not be perceived as such.

The suprachiasmatic nucleus (SCN) is the pacemaker of the mammalian circadian timing system and regulates daily cycles in activity, hormonal levels, and other physiological variables (Moore and Eichler, 1972; Stephan and Zucker, 1972). The primary input of the SCN is light, and this pathway possibly plays an important role in acute alerting effects of light (Cajochen et al., 2000).

A high level of alertness is defined as a state of high sensitivity to incoming stimuli (Posner, 2008). It is known to affect many psychological and physiological functions, such as performance, caloric intake, and pain sensitivity (Alexandre et al., 2017; Curcio et al., 2001; Figueiro et al., 2015; Pardi et al., 2016). White light has been shown to significantly improve alertness during the evening and night (Cajochen et al., 2000). However, effects during daytime are less conclusive (reviewed in Lok et al., 2018a). Together, these studies implicate differences in alerting effects of light at different times of day.

Human alertness decreases with wakefulness duration, but also fluctuates independent of accumulated sleep pressure, with a cycle of approximately 24 hours (Wright et al., 2002). This indicates an influence of the SCN (Aston-Jones, 2005). The circadian drive for alertness increases during the day when homeostatic sleep pressure levels increase, resulting in relatively stable levels of alertness during the wake period (Dijk et al., 1992; Wright et al., 2002; Wu et al., 2015; Wyatt et al., 2006; Zhou et al., 2011a, 2011b). Discrepancies in alerting effects of light reported by studies investigating these effects at different times of day suggest that light effects on alertness might be modulated by circadian clock phase and/or by the amount of accumulated sleep pressure.

One approach to distinguish circadian variation from wake duration related variation is a forced desynchrony (FD) paradigm. In FD paradigms, wake and sleep periods are scheduled with a duration that deviates sufficiently from 24 h such that it falls outside the range of circadian entrainment by light. This allows the internal pacemaker to free run (i.e., following its intrinsic circadian period) throughout scheduled sleep and wakefulness (Kleitman and Kleitman, 1953). As a consequence, sleep and wake intervals are scheduled at different circadian phases, resulting in multiple combinations of homeostatic sleep drive levels and circadian phases along the FD protocol. Under certain assumptions, it is possible to mathematically disentangle wake duration related and circadian clock phase effects on parameters of interest (Dijk et al., 1992; Hiddinga et al., 1997).

Most FD protocols have only been performed in dim light (<15 lux), with the exception of Zitting et al. (2018). Light is known to phase shift the SCN, which can interfere with methods of disentangling wake duration related variation from circadian components. This is partially caused by non-uniform phase progression of the circadian system with the sleep-wake pattern and synchronous light-dark cycle (also called relative coordination (Wever, 1977)). However, examination of the parameter space of the human circadian pacemaker indicated the possibility to run a FD design at 1300 lux without significant effects of relative coordination (Woelders et al., 2017). To investigate the contribution of both circadian and wake duration related variation to alerting effects of light, we conducted such an FD experiment in humans under both low and high light intensities. We hypothesize that alerting effects of light will depend on both wake duration related variation and circadian clock phase. Given previously reported discrepancies between objective and subjective measures of alertness, light effects will probably not be similar for subjective and objective measures (Zhou et al., 2012). To our knowledge, bright light FDs have not been previously conducted, the argument being that bright light induces a non-uniform progression of circadian phase. We show with model simulations that a 13 h wake 5 h sleep alternation yields uniform phase progression both in dim light and under 1300 lux of bright light. We hypothesized that this would also be the case in such forced desynchrony study.

## Materials and Methods

### Power Calculation

Ruget al. (2005) reported an effect size of 1.9 on the Karolinska Sleepiness Scale after 1 h of bright light exposure effect size of 1.9 on the Karolinska Sleepiness Scale was found after 1 h of bright light exposure. With alpha set to 0.05 and power to 0.8, a total of 5 participants per light condition should be included to find a statistically significant difference on the Karolinska Sleepiness Scale. To ensure sufficient power on other output parameters that are likely less sensitive, 8 participants were included (Lok et al., 2018a).

### Subjects

Participants were healthy, non-sleep deprived males (n=8) between 20 and 30 years of age (average ± SEM: 24.0 ± 1.16). Participants provided written informed consent and received financial compensation for participation. Study protocol, screening questionnaires, and consent forms were approved by the medical ethics committee of the University Medical Center Groningen (NL54128.042) and were in agreement with the Declaration of Helsinki (2001).

An in-house developed general health questionnaire was used to assess health of the participants. As an indication of sleep timing, chronotype was assessed via the Munich Chronotype Questionnaire (MCTQ; Roenneberg et al., 2003). To determine baseline sleep quality, participants completed the Pittsburgh Sleep Quality Index (PSQI; Buysse et al., 1989). Participants reported no health problems, were intermediate chronotypes (Midpoint of sleep on work-free days, sleep-corrected [MSF_sc_] average ± SEM: 4.88 ± 0.60), and did not report more than mild sleep problems (average ± SEM: 4.63 ± 1.13). Exclusion criteria were (1) chronic medical conditions or the need for medication use, (2) shift work within 3 months before participation, (3) having traveled over multiple time zones within 2 months before participation, (4) smoking, (5) moderate to high levels of caffeine intake (>4 cups per day, estimated average ± SEM was 0.75 ± 0.35 cups per day for included participants), (6) excessive alcohol use (>3 glasses per day), (7) use of recreational drugs in the last year, (8) body mass index outside the range of 18 to 27, (9) inability to complete Ishihara color blindness test (Ishihara, 1972) without errors upon arrival. Two participants reported to never drink alcohol, 2 others drank 1 to 2 glasses a month, and 4 participants reported drinking 2 to 3 glasses a week.

### Protocol

**Figure 1.**
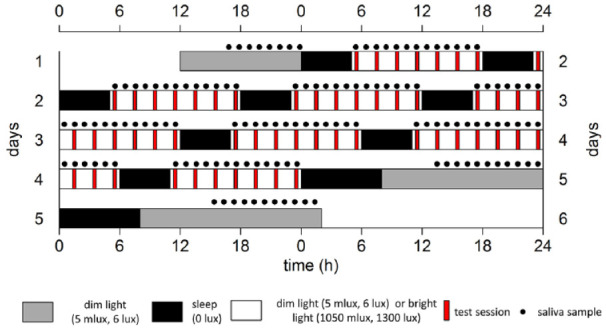
Schematic representation of the experiment design, for an individual with a habitual sleep onset at 00:00 h, double-plotted. Gray bars indicate dim light (6 lux) conditions preceding/following the forced desynchrony protocol. Black bars represent intervals for sleep (5 h, except for the last sleep attempt which is allowed to last for 8 h) with lights off (0 lux), while white bars represent wakefulness in either polychromatic white dim light (6 lux, 5 melanopic lux) or bright light (1300 lux, 1050 melanopic lux) conditions. Black dots indicate saliva samples for melatonin and cortisol assessments, red bars indicate test sessions. After completion of a test session, subject received an iso-caloric snack. The protocol lasted for 72 h, therefore comprising a full beat (3 × 24 h = 72 h, 4 × 18 h = 72 h). Color version of the figure is available online.

Subjects were equipped with wrist worn Actigraphy (CamNtech, United Kingdom) 5 weeks before the start of the in-lab FD experiment to monitor regularity in sleep/wake cycles. On the first day of the lab protocol, participants arrived at the human isolation facility of the University of Groningen 10 h before habitual sleep onset (hson; assessed with the MCTQ). Upon arrival, individuals were equipped with EEG electrodes at the left and right frontal, central and occipital locations, electro-oculogram electrodes placed above and below both eyes, and 2 electromyography electrodes underneath the chin. Reference electrodes were placed on the left and right mastoid. Dim light melatonin onset (DLMO) was assessed through hourly saliva samples, from 7 h before hson onward. After the last saliva sample, the FD protocol started with 5 h for sleep. Participants were woken up under polychromatic white light of either dim (DL, 6 lux) or bright light intensity (BL, 1300 lux), both intensities measured vertically at the level of the eye (for more specifications of the light, see paragraph below). Subjects remained awake under these light conditions for the next 13 h (Figure 1). Two-hourly test sessions were performed during wakefulness, starting 20 min after awakening. During wakefulness, saliva samples to determine melatonin and cortisol concentrations were taken hourly. Iso-caloric snacks were provided immediately after completion of each test session, with caloric value based on estimated basic metabolic rate, according to the following equation: BMR = ((10 × weight(kg)) + (6.25 × length(cm)) – (5 × age(years)) + 5)) (Mifflin et al., 1990). After 13 h of wakefulness under dim or bright light, participants were instructed to go to bed, after which the light was switched off. The 18 h FD cycle, consisting of 5 h for sleep and 13 h for wakefulness, was repeated 4 times, resulting in a 72 h forced desynchrony protocol (4 times 18 h exactly matches 3 times 24 h). After completion, subjects were offered an additional sleep opportunity of 3 h. They remained in dim light in the human isolation facility. From 9 h before the following hson until 2 h after that hson, saliva samples for DLMO determination were collected. After the last sample, subjects were allowed to go home while wearing wrist Actigraphy and returned after at least 3 weeks to participate in the same protocol under opposite light conditions. The order of light conditions was counterbalanced. The experiment was conducted between February and May 2018.

### Mathematical Simulations

A modified version of Kronauer’s model of the human circadian pacemaker (Woelders et al., 2017) was used to model clock phase changes during a 72 h (4 × 18 h) forced desynchrony (5 h sleep – 13 h wakefulness) under dim light (DL; 5 melanopic lux, 6 lux) and bright light (BL; 1050 melanopic lux, 1300 lux) conditions. Simulations with intrinsic circadian periods (τ) ranging from 24 to 24.6 h indicated a free running rhythm of the circadian pacemaker with uniform phase progression under both light conditions (Suppl. Fig. S1). Further simulations indicated that 5 h of sleep and 13 h of wakefulness minimized chances of non-uniform phase progression, since with this ratio, light exposure is timed in such a way that both the delay and advance zone of the phase response curve are hit equally (Suppl. Fig. S1). Simulations with the 2-process model of sleep regulation (Daan et al., 1984) allowed estimation of sleep pressure build-up under different sleep-wake durations. Accumulated sleep pressure at the end of wakefulness after 5 h sleep–13 h wakefulness was similar to a 8 h sleep−16 h wake cycle (Suppl. Fig. S2). According to the model, sleep pressure levels did not systematically increase or decrease over the course of the FD protocol (Suppl. Fig. S2).

### Test Session

Test sessions started with EEG recordings (Åkerstedt and Gillberg, 1990), in which alpha and theta power density during eyes-closed (2 min) and open (6 min, while focusing on a fixation mark) were determined. Thereafter, subjects completed the Karolinska Sleepiness Scale (Åkerstedt and Gillberg, 1990), which was followed by a 5 min auditory Psychomotor Vigilance (PVT) and a 5 min auditory Go-NoGo (GNG) task, to assess sustained attention (Dinges and Powell, 1985) and executive control (Barry et al., 2014), respectively.

### Light Exposure

Polychromatic white DL and BL were provided via ceiling-mounted Philips fluorescent light tubes (see Suppl. Table S1 and Fig. S3 for α-opic illuminance values and spectral composition). Each participant was exposed to the same light intensity throughout the light phase of one FD protocol.

### Hormone Analysis

Saliva was collected using Sarstedt Salivettes with a cotton swab (Salivette, Sarstedt BV, Etten-Leur, the Netherlands). Melatonin concentrations were assessed by radioimmunoassay (RIA) (RK-DSM2; Bühlmann Laboratories AG, Schönenbuch, Switzerland). The limit of detection was 0.5 pg/ml with 9.1% intra-assay variation, and 18.1% inter-assay variation in lowest concentrations and 14.1% in highest concentrations. Cortisol concentrations were detected with RIA analysis (CORT-C2; Cisbio, Bioassays, Parc Marcel Boiteux, Codolet, France), including Bio-rad Immunoassay Plus Control (Lyphochek). The limit of detection was 1 nmol/l, with 10.4% intra-assay and 9.7% inter-assay variation at low and 3.4% at high cortisol concentrations.

### Tau Estimation

Tau was estimated by melatonin data, measured as DLMO (defined as crossing of the 3 pg/ml concentration, by linear interpolating of raw values before and after the cutoff point) preceding and following the FD protocol. For one individual, tau could not be assessed due to missing DLMO, resulting in *n* = 7 subjects.

### PVT and GNG

If a response occurred before the time calculated as the average response time of all test sessions minus two standard deviations, it was defined as an anticipation error. Errors of omission were responses occurring after the time calculated as the average of all test sessions plus two standard deviations. These definitions are the preferred method over a set cutoff point, since it yields a more accurate representation of individually varying reaction times (Gabel et al., 2019). Nevertheless, to compare results presented here to literature, a cutoff point of 500 ms was also employed. In the GNG task, errors of commission were characterized as responding to a non-response tone. Other parameters of interest were averages of the 10% fastest and 10% slowest reaction times.

### EEG-Based Indices of Alertness

EEG data were collected using the Temec EEG (Vitaport, 28 channels) system. Electrode impedance was maintained below 5 kΩ at all 6 electrode locations (O3, O4, C3, C4, F3, F4). Data were analyzed using Vitascore V1.60. Artifacts were manually removed, and high-pass (0.5 Hz) and low-pass (30 Hz) digital filters were applied. Based on the Karolinska Drowsiness Test, alpha (8.0–12.0 Hz) and theta (4.0–7.9 Hz) power values during eyes-closed and eyes-open were calculated using Fast Fourier transform (FFT) with a 4 s bin width (Åkerstedt and Gillberg, 1990). Power spectra were calculated for every 30 s epoch of EEG data on every derivate.

### Statistics

RStudio (version 1.0.136) was used for statistics and graphics. Wake duration related variation in performance and subjective alertness were quantified by grouping and averaging data in 2 h bins according to time since sleep offset, starting 0.5 h after waking until 12 h later. Wake duration related regulation of melatonin and cortisol were quantified in hourly bins. To determine circadian variation, original data were calculated as a function of circadian phase (in degrees relative to DLMO) in 30 degree bins. For optimal clarity, corresponding time of day (h) is depicted for both wake duration related and circadian variation for an individual with wake-up time of 08:00 and a DLMO of 19:00. Linear mixed models were constructed with light condition as independent variable, time since sleep offset and circadian phase as a fixed effect (categorical variable) and added interaction terms between time since sleep offset and light, and circadian phase and light condition. Subject ID and visit were included as random effects to control for between subject variation and possible order effects. Critical 2-sided significance level alpha was 0.05 for all statistical tests. To ensure sufficient sample size (*n* ≥ 3) for each combination of “time since sleep offset” and “circadian clock phase,” we constructed a separate linear mixed model to calculate significance of the interaction terms of these variables with light condition. Contrast analyses (comprising of a Tukey post hoc test corrected for multiple testing [Tuckey correction], package “lsmeans”) was conducted on all combinations of circadian time and time since sleep offset. Contrasts were constructed in 60 degree bins, with wake-dependent changes in bins of 2 h for melatonin and cortisol concentrations or 4 h for alertness parameters. Significant contrasts are depicted in 3-dimensional graphs, in which circadian variation (in 60 degree bins) is depicted on the x-axis, wake duration related variation on the y-axis, and BL scores were subtracted from DL scores, with colors indicating the direction and magnitude of the light effect. Combinations of wake duration related variation and circadian variation that contain data of fewer than 4 individuals are considered “missing data” and are depicted in grey. To estimate light effects during the time course over the regular day, sleep was predicted to start 2 h after DLMO (Burgess et al., 2003) (coinciding with circadian phase 30) and last for 5 h (until circadian phase 160), after which 13 h of wakefulness commenced. Whether light effects occurred during the projected daily time course was visually assessed.

## Results

### Melatonin and Cortisol

**Figure 2.**
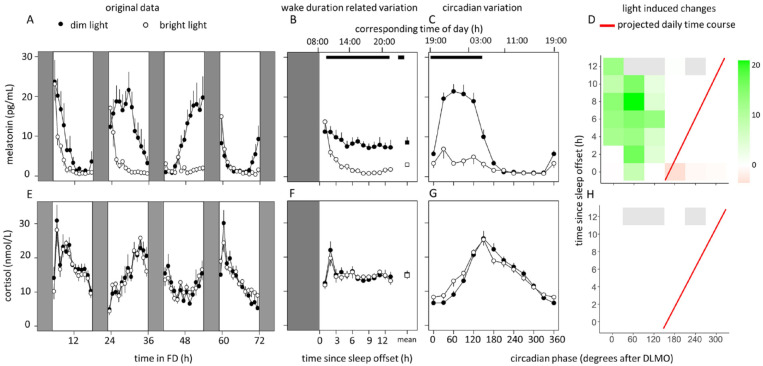
Data of melatonin (top panel) and cortisol (bottom panel) concentrations. Time course of melatonin (a) and cortisol (e) during the FD protocol. Data replotted as time since sleep offset (b and f) and circadian phase in degrees after DLMO (phase 0, c and g), for melatonin and cortisol respectively. Corresponding time of day (h) is depicted on the top axis. Contrast analysis describing light-induced decrease for all combinations of circadian clock phase and time since sleep offset for melatonin (d) and cortisol (h). Data represent mean ± standard error of the mean, with 7 subjects per group. Black dots indicate data collected in dim light, white dots represent data collected in bright light and black and white squares represent averages over all data points under dim light and bright light, respectively. Red line indicates the expected time course over a regular day. Shaded areas represent scheduled sleep (at 0 lux). Significant differences between light conditions (*p* < 0.05) are indicated by horizontal black bars (b, c, f, g) or colored rectangles (d, h). Gray rectangles indicate combinations of wake duration related variation and circadian clock phase containing data of less than 4 individuals (d, h). Abbreviations: FD = forced desynchrony; DLMO = dim light melatonin onset. Color version of the figure is available online.

Tau increased significantly (on average 21 min) after BL exposure (DL, average ± SEM: 24.25 ± 0.09; BL, average ± SEM: 24.60 ± 0.11, *p* = 2·10^-4^, Suppl. Fig. S4). Melatonin rhythms showed robust oscillations in DL, but disrupted rhythms in BL due to light-induced melatonin suppression (Figure 2a and Table 1). There was an effect of time awake on melatonin levels, with significant differences between light conditions (Figure 2b, Table 1). Significant differences between BL and DL also existed depending on circadian phases, showing dampened melatonin rhythms in BL, but significant variation in DL (Figure 2c, Table 1). These effects were even more apparent in 3D plots, with significantly higher melatonin values after DLMO (phase 0) in the DL condition, without significant differences at circadian phases when melatonin was absent (Figure 2d).

**Table 1.** Summary of statistics of wake duration related variation (process S), circadian variation (process C), interaction between process S and light, C and light, and additive effects of bright light exposure.

	Wake duration related variation (process S)		Circadian variation (process C)		Interaction (process S × light)		Interaction (process C × light)		Additive effect of bright light	
Melatonin	*F*_(12, 672),_ *p*	**10.87,** **<** **2.20 x 10_-16_**	*F*_(5, 672),_ *p*	**56.41,** **<** **2.20 x 10_-16_**	*F*_(12, 672),_ *p*	**5.15,** **<** **3.12 x 10_-8_**	*F*_(11, 672),_ *p*	**24.73,** **<** **2.20 x 10_-16_**	*F*_(1, 672),_ *p*	**5199.52,** **<** **2.20 x 10_-16_**
Cortisol	*F*_(12, 672),_ *p*	**3.95,** **<** **7.20 x 10_-6_**	*F*_(5, 672),_ *p*	**22.18,** **<** **2.20 x 10_-16_**	*F*_(12, 672),_ *p*	0.22, > 0.05	*F*_(11, 672),_ *p*	0.38, > 0.05	*F*_(1, 672),_ *p*	0.01, > 0.05
Subjective alertness	*F*_(6, 392),_ *p*	**17.87,** **<** **2.20 x 10_-16_**	*F*_(5, 392),_ *p*	**2.54,** **<** **4.40 x 10_-3_**	*F*_(12, 392),_ *p*	1.38, > 0.05	*F*_(11, 672),_ *p*	1.03, > 0.05	*F*_(1, 392),_ *p*	2.27, > 0.05
PVT reaction time	*F*_(6, 392),_ *p*	0.53, > 0.05	*F*_(5, 392),_ *p*	0.77, > 0.05	*F*_(12, 392),_ *p*	0.31, > 0.05	*F*_(11, 672),_ *p*	0.32, > 0.05	*F*_(1, 392),_ *p*	**104.93,** **<** **2.20 x 10_-16_**
GNG reaction time	*F*_(6, 392),_ *p*	0.79, > 0.05	*F*_(5, 392),_ *p*	0.54, > 0.05	*F*_(12, 392),_ *p*	0.36, > 0.05	*F*_(11, 672),_ *p*	0.42, > 0.05	*F*_(1, 392),_ *p*	**88.16,** **<** **2.20 x 10_-16_**

Abbreviations: PVT = Psychomotor Vigilance; GNG = Go-NoGo. Values from linear mixed models on melatonin and cortisol concentrations, subjective alertness scores, and performance (PVT and GNG).

Successive cortisol cycles indicate free running of the circadian clock, without significant differences between light conditions (Figure 2e, Table 1). There were significant effects of time awake (Figure 2f, Table 1). Cortisol levels varied significantly with circadian phase, independent of light condition (Figure 2 g, Table 1). There were no significant interaction effects between time and light condition (Figure 2 h).

### Relative Coordination

To investigate possible relative coordination, residual cortisol data were analyzed after subtraction of the calculated wake duration related variation and circadian variation. Results indicated absence of significant relative coordination, since a fitted 72 h sine wave was non-significant (DL: *F*_2,359_ = 0.14, *p* = 0.87 and BL: *F*_2,359_ = 0.17, *p* = 0.84; Suppl. Fig. S5). The residual variation was respectively 1.61% and 1.83% of the variation in the raw data under DL and BL conditions, indicating that the calculated homeostatic and circadian phase fluctuations explain almost all of the variation in cortisol concentrations.

### Subjective Alertness

**Figure 3.**
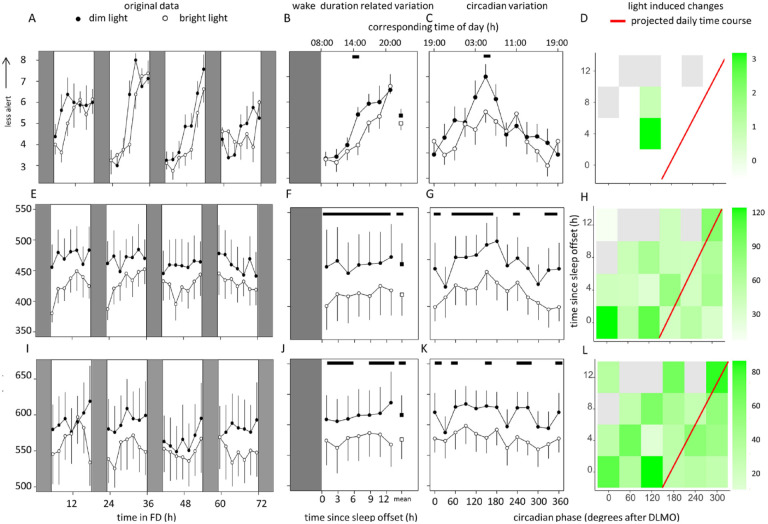
Data of subjective alertness (top panel), average reaction time on the PVT (middle panel) and GNG (bottom panel). Time course of subjective alertness (a), performance on the PVT (e), and GNG (i) during the FD protocol. Data replotted as time since sleep offset (b, f, and j) and circadian phase in degrees after DLMO (phase 0, c, g, and k), for subjective alertness, PVT performance, and GNG performance, respectively. Corresponding time of day (h) is depicted on the top axis. Contrast analysis describing light-induced decrease for all combinations of circadian clock phase and time since sleep offset for subjective sleepiness (d), PVT performance (h), and GNG performance (l). Data represent mean ± standard error of the mean, with 7 subjects per group. Black dots indicate data collected in dim light, white dots represent data collected in bright light, and black and white squares represent averages over all data points under dim light and bright light, respectively. Red line indicates the expected time course over a regular day. Shaded areas represent scheduled sleep (at 0 lux). Significant differences between light conditions (*p* < 0.05) are indicated by horizontal black bars (b, c, f, g, j, k) or colored rectangles (d, h, l). Gray rectangles indicate combinations of wake duration related variation and circadian clock phase containing data of less than 4 individuals (d, h). Abbreviations: PVT = Psychomotor Vigilance; GNG = Go-NoGo; FD = forced desynchrony; DLMO = Dim light melatonin onset. Color version of the figure is available online.

Subjective alertness scores (Figure 3a) increased with time awake, and subjective sleepiness increased later under BL compared with DL conditions (Figure 3b). Circadian variation was established, independent of light exposure (Figure 3c, Table 1). Significant interactions were found between circadian phase, time since sleep offset, and light-induced change in subjective alertness, with increased alertness predominantly after DLMO (phase 0). The lack of statistically significant effects appears to be present during the projected daily time course (Figure 3d).

### Performance: PVT

Sustained attention (defined as average reaction time on the PVT [ms], Figure 3e) was better in BL compared to DL, independent of time awake (Figure 3f, Table 1). There was no significant effect of circadian phase, but there were significant differences between light conditions (Figure 3 g, Table 1). Significant interactions were found between circadian phase, time since sleep offset, and light-induced change in performance. Significant light-induced improvements in reaction time also occurred during the projected daily time course (Figure 3 h). Detailed parameters of the PVT task can be found in Supplementary Figure S6, Table S2.

**Table 2.** Summary of effects of wake duration related variation, internal clock time, the interaction between both processes, direction of additive effects of bright light exposure, and interaction effects between light exposure and timing of exposure during the daily time course.

	Wake Duration Related Variation (Process S)	Internal Clock Time (Process C)	Interaction (Process S × C)	Additive Effect of Bright Light	Interactive Effect During Daily Time Course
Melatonin	Y	Y	Y	–	N
Cortisol	Y	Y	Y	0	N
Subjective alertness	Y	Y	N	0	N
PVT reaction time	N	N	N	–	Y
GNG reaction time	N	N	N	–	Y

“Y” and “N” indicate significant or non-significant effects respectively, “+” indicates a significant increase, “–” represents a significant decrease, and non-significant changes are indicated by “0.”

Abbreviations: PVT = Psychomotor Vigilance; GNG = Go-NoGo.

### Performance: GNG

Executive performance (assessed with the GNG task, Figure 3i) was better in BL independent of time awake (Figure 3j, Table 1). There was no significant effect of circadian phase, which was affected by light exposure (Figure 3k, Table 1). There were significant interactions between circadian phase, time since sleep offset, and light-induced change in performance. Significant light-induced improvements in reaction time also occurred during the projected daily time course (Figure 3l). Detailed parameters of this task can be found in Supplementary Figure S7, Table S2. All results are summarized in Table 2.

EEG-based correlates of alertness can be found in Supplementary Figures S8-S10 and Table S3. Correlations between alertness measures can be found in Supplementary Figure S11.

## Discussion

Light can increase (subjective) alertness during the night, but results are less conclusive during daytime (Lok et al., 2018a). Both wake duration related variation and circadian clock phase might contribute to this discrepancy. In the current FD study, we found that light can increase performance independent of time awake, while positive effects of light on subjective alertness are only reported 6 to 9 h after waking up. High-intensity light exposure effectively postponed the increase of subjective sleepiness with progressing time awake, although light-induced improvements could not be determined (through visual assessment) during the projected daily time course. Performance was better throughout the wake interval in high-intensity light, independent of sleep pressure build-up or circadian clock phase, with significant improvements occurring during the projected daily time course.

### Bright Light FD Design and Cortisol as a Phase Marker

Dim light (<10 lux) has been standard procedure since the first FD experiment was performed (Kleitman and Kleitman, 1953). Apart from model calculations (Woelders et al., 2017), this is the first study we know showing that short FD protocols can also be used for disentangling effects of bright light gated by wake duration related variation and circadian processes. During FD paradigms, concentrations of the nocturnal hormone melatonin have regularly been used as output measures of SCN rhythmicity. Cortisol had to be used as circadian phase marker during this experiment, because high-intensity light suppresses melatonin production (Rollag et al., 1980). A free-running 24 h cortisol rhythm with uniform phase progression in both light conditions suggests that cortisol can be used as a reliable output measure of the circadian clock under these controlled conditions. Suggestions that light might decrease (Beck-Friis et al., 1985; Jung et al., 2010; Kostoglou-Athanassiou et al., 1998) or increase (Scheer et al., 1999) cortisol in humans are not confirmed by our data and others (Lavoie et al., 2003; Mcintyre et al., 1992; Ruger et al., 2005). The absence of the cortisol awakening response in combinations of circadian clock phases and wake duration related variation employed in the current FD protocol may have contributed to the absence of light effects on cortisol. At least 54 FD studies have shown uniform phase progression under DL conditions (Archer et al., 2014; Beersma and Hiddinga, 1998; Bermudez et al., 2016; Boivin et al., 1997; Burgess and Eastman, 2008; Burke et al., 2015; Buxton et al., 2012; Cain et al., 2007; Cajochen et al., 2002; Carskadon et al., 1999; Czeisler et al., 1999; Darwent et al., 2010; Dijk and Czeisler, 1994; Dijk and Edgar, 1999; Dijk et al., 1992, 1999; Duffy et al., 2001, 2011; Ferguson et al., 2012; Gronfier et al., 2007; Hiddinga et al., 1997; Hilton et al., 2000; Hu et al., 2004; Hull et al., 2003; Ivanov et al., 2007; Johnson et al., 1992; Kendall et al., 2001; Klerman et al., 1998; Kline et al., 2010; Koorengevel et al., 2000, 2003; Kosmadopoulos et al., 2014; Kripke et al., 2005; Lazar et al., 2015; Lee et al., 2009; Monk and Carrier, 1998; O’Donnell et al., 2009; Postnova et al., 2016; Sargent et al., 2012; Scheer et al., 2009, 2010, 2011; Shea et al., 2011; Silva et al., 2010; Waterhouse et al., 2004; Wright et al., 2002; Wright et al., 2005, 2006; Wu et al., 2015; Wyatt et al., 1999, 2004, 2006; Zhou et al., 2011a, 2011b). Since the SCN is light sensitive (Klein et al., 1991; Ralph et al., 1990), it can be expected that phase progression is less uniform in BL. If phase progression would be non-uniform in BL but uniform in DL, then cortisol concentrations would differ at sections of the cortisol curves. 3D analysis, in which cortisol concentrations between DL and BL conditions were compared point by point, indicate no significant differences between the two light conditions (Figure 2 h). Hence, there is no evidence for non-uniform phase progression under BL in this FD protocol. We cannot exclude the possibility that there is non-uniform phase progression in other tissues that are under the influence of light, and that have not been measured in our FD protocol. For instance, firing rates of the SCN could be affected, with possible phase shifts as a result. Although light responsive parameters measured in this protocol do not show such reduction in circadian amplitude, we cannot exclude the possibility that changes in firing rates do occur. Nonetheless, this study presents evidence that FD designs can be conducted under high-intensity lighting.

### Does Bright Light Induce Lengthening of Internal Period?

Based on the dim light melatonin onset preceding and following the FD protocol, the internal circadian period showed a 21 min increase under BL, suggesting a lengthening of the internal period (Suppl. Fig. S4). This is in line with data observed in most mammals, including diurnal primates, lengthening showing an increase in circadian period under continuous light exposure (Klerman et al., 1996; Weber, 1967). On the other hand, diurnal vertebrates overall seem to decrease circadian period under the influence of light, as was described by Aschoff’s rule and his own findings in humans (Aschoff, 1958, 1965). Earlier model simulations indicated that FD protocols with limited amount of cycles may typically overestimate circadian period at high light intensities (Woelders et al., 2017), and should therefore not be over-interpreted.

### Subjective Alerting Effects of Light Depend on Homeostatic Control

Our data confirm subjective alertness increases with wakefulness duration (Dijk et al., 1992). Subjective alertness ratings were similar under both light conditions, both at the start and the end of the light phase, suggesting that the decrease in alertness is postponed by high-intensity light exposure. Subjective alertness scores after awakening were similar to natural conditions (Åkerstedt et al., 2017), suggesting that 5 h for sleep, 13 h wakefulness did not increase sleep pressure levels. Moreover, the relationship between alertness scores and alerting effects of light based on homeostatic sleep pressure suggest a parabolic function; a ceiling effect is present after awakening, and as a consequence alertness cannot improve beyond its limits, but with increasing sleep pressure build-up, alertness decreases, creating room for improvement by light. When sleep pressure rises further, accumulated sleep pressure overrides positive effects of bright light exposure. This hypothesis is consistent with the observations that alerting effects of light during daytime are hard to determine in well-rested individuals (Lok et al., 2018b), while significant effects have been reported in sleep deprived individuals (Phipps-Nelson et al., 2003). In addition to effects of time awake, there is also circadian variation in subjective alertness, with the largest decrease in alertness at the end of the night, as has been reported in other FD studies (Dijk et al., 1992). Most likely, this contributes to sleep maintenance when most sleep pressure has dissipated (Dijk and Czeisler, 1994; Dijk et al., 1992). Likewise, the circadian contribution to alertness at the end of the day compensates for the increased homeostatic sleep pressure at that time.

### Light-Induced Performance Improvements Are Independent of Homeostatic Control

Performance was significantly improved under bright light, but did not change as a function of time of day. This is in contrast with some (Silva et al., 2010; Wright et al., 2002) but not all literature (Lee et al., 2009; Zhou et al., 2011a), in which significant effects of time awake have been described. Similar patterns have been reported in EEG-based indices of alertness (Cajochen et al., 2002). A possible explanation for discrepancies between our and other studies might be varying lengths of FD protocols. Longer FD cycles induce higher sleep pressure levels and therefore larger declines in performance and increases in alpha-theta activity. Moreover, accumulating sleep pressure may differently affect subjective alertness, performance, and EEG (Postnova et al., 2018), as these measures do not always correlate well together (Leproult et al., 2003; Rahman et al., 2014; Van Dongen et al., 2003; Zhou et al., 2012). Our data add to the body of literature supporting this, since both performance and wake EEG reflect similar patterns, which do not coincide with subjective alertness patterns (Suppl. Fig. S11). It is noteworthy that significant improvements in performance were present after merely 20 min of high-intensity light exposure on the first day of the FD paradigm, suggesting that light effects on these parameters are relatively fast. Light exposure may counteract initial decrements in performance due to sleep inertia after awakening. The significant improvement of alertness by light in the middle of the subjective night (circadian phase 150) might be considered to be in line with findings of light-induced improvements in the early night under entrained conditions (Cajochen et al., 2005).

### Limitations

First, financial and physiological limitation forced us to conduct this study in male participants only. Since body temperature, that is known to influence sleep timing (Murphy and Campbell, 1997), fluctuates more in female (Driver et al., 1996; Kolka and Stephenson, 1989) than male participants, this study only uses male participants. This clearly hinders extrapolation to other genders. Future studies should employ both genders to verify that conclusions presented in this article also account for females. Second, the relatively low number of participants could complicate statistical interpretation. However, given (1) that power calculations show that significant light effects on subjective sleepiness can already be found with merely 5 participants per light condition and (2) we report highly significant findings (Table 1), this seems unlikely. Third, the protocol duration is relatively short, encompassing merely one beat cycle. Although multiple beat cycles are desirable, a short FD design is necessary to prevent extensive light-induced tau elongations (Suppl. Fig. S4), which could complicate comparison between the DL and BL conditions. Fourth, the chosen sleep:wakefulness ratio is 1:3, deviating from the classical 1:2 ratio. There are several reasons for this sleep-wake ratio: (1) Since uniform phase progression is essential for FD protocols, the 5:13 ratio had to be chosen, as this was the only combination that ensured equal stimulation of the phase delay and advance part of the phase response curve (Suppl. Fig. S1). (2) In addition, given this protocol encompasses merely one complete beat, the 5:13 ratio ensured sleep data at all clock phases, even when the circadian system promoted wakefulness. (3) The primary goal of this study was to compare bright versus dim light effects on human alertness, and under both protocols the 5:13 sleep to wakefulness ratio was used. Light effects are therefore still valid. Furthermore, simulations with the 2-process model of sleep show that homeostatic sleep pressure levels do not systematically increase or decrease over the 72 h of this forced desynchrony protocol (Suppl. Fig. S1).

In conclusion, this is the first study to investigate effects of light on alertness under forced desynchrony conditions, showing (1) stable free-running cortisol rhythms with uniform phase progression under both light conditions, suggesting that FD designs can be conducted under high-intensity lighting, (2) that bright light can postpone the onset of subjective alertness, depending on wake duration induced variation, and (3) that performance is improved by high-intensity light independent of wake duration related variation or circadian clock phase. Data collected here indicate that during the projected time course of a regular day, light can improve objective, but not subjective parameters of alertness. This suggests that high-intensity indoor light exposure during office hours might be beneficial for mental performance, even though this may not be perceived as such.

## Supplemental Material

sj-docx-1-jbr-10.1177_07487304221096945 – Supplemental material for Bright Light Increases Alertness and Not Cortisol in Healthy Men: A Forced Desynchrony Study Under Dim and Bright Light (I) Click here for additional data file. Supplemental material, sj-docx-1-jbr-10.1177_07487304221096945 for Bright Light Increases Alertness and Not Cortisol in Healthy Men: A Forced Desynchrony Study Under Dim and Bright Light (I) by R. Lok, T. Woelders, M. J. van Koningsveld, K. Oberman, S. G. Fuhler, D. G. M. Beersma and R. A. Hut in Journal of Biological Rhythms

## References

[bibr1-07487304221096945] Åkerstedt T Gillberg M (1990) Subjective and objective sleepiness in the active individual. Int J Neurosci 52:29-37. 2265922 10.3109/00207459008994241

[bibr2-07487304221096945] Åkerstedt T Hallvig D Kecklund G (2017) Normative data on the diurnal pattern of the Karolinska Sleepiness Scale ratings and its relation to age, sex, work, stress, sleep quality and sickness absence/illness in a large sample of daytime workers. J Sleep Res 26:559-566. 28370590 10.1111/jsr.12528

[bibr3-07487304221096945] Alexandre C Latremoliere A Ferreira A Miracca G Yamamoto M Scammell TE Woolf CJ (2017) Decreased alertness due to sleep loss increases pain sensitivity in mice. Nat Med 23:768-774. 28481358 10.1038/nm.4329 PMC5798598

[bibr4-07487304221096945] Archer SN Laing EE Möller-Levet CS van der Veen DR Bucca G Lazar AS Santhi N Slak A Kabiljo R von Schantz M , et al. (2014) Mistimed sleep disrupts circadian regulation of the human transcriptome. Proc Natl Acad Sci U S A 111:E682-E691. 24449876 10.1073/pnas.1316335111 PMC3926083

[bibr5-07487304221096945] Aschoff J (1958) Tierische Periodik unter dem Einfluss von Zeitgebern. Z Tierpsychologie 15:1-30.

[bibr6-07487304221096945] Aschoff J (1965) Circadian rhythms in man. Science 148:1427-1432. 14294139 10.1126/science.148.3676.1427

[bibr7-07487304221096945] Aston-Jones G (2005) Brain structures and receptors involved in alertness. Sleep Med 6:3-7. 10.1016/s1389-9457(05)80002-4 16140243

[bibr8-07487304221096945] Barry RJ De Blasio FM De Pascalis V Karamacoska D (2014) Preferred EEG brain states at stimulus onset in a fixed interstimulus interval equiprobable auditory Go/NoGo task: a definitive study. Int J Psychophysiol 94:42-58. 25043955 10.1016/j.ijpsycho.2014.07.005

[bibr9-07487304221096945] Beck-Friis J Borg G Wetterberg L (1985) Rebound increase of nocturnal serum melatonin levels following evening suppression by bright light exposure in healthy men: relation to cortisol levels and morning exposure. Ann N Y Acad Sci 453:371-375.

[bibr10-07487304221096945] Beersma DGM Hiddinga AE (1998) No impact of physical activity on the period of the circadian pacemaker in humans. Chronobiol Int 15:49-57. 9493714 10.3109/07420529808998669

[bibr11-07487304221096945] Bermudez EB Klerman EB Czeisler CA Cohen DA Wyatt JK Phillips AJK (2016) Prediction of vigilant attention and cognitive performance using self-reported alertness, circadian phase, hours since awakening, and accumulated sleep loss. PLoS ONE 11:e0151770. 27019198 10.1371/journal.pone.0151770 PMC4809494

[bibr12-07487304221096945] Boivin DB Czeisler CA Dijk DJ Duffy JF Folkard S Minors DS Totterdell P Waterhouse J (1997) Complex interaction of the sleep-wake cycle and circadian phase modulates mood in healthy subjects. Arch Gen Psychiatry 54: 145–152. 10.1001/archpsyc.1997.01830140055010 9040282

[bibr13-07487304221096945] Burgess HJ Eastman CI (2008) Human tau in an ultradian light-dark cycle. J Biol Rhythms 23:374-376. 18663244 10.1177/0748730408318592 PMC3622149

[bibr14-07487304221096945] Burgess HJ Savic N Sletten T Roach G Gilbert SS Dawson D (2003) The relationship between the dim light melatonin onset and sleep on a regular schedule in young healthy adults. Behav Sleep Med 1:102-114. 15600132 10.1207/S15402010BSM0102_3

[bibr15-07487304221096945] Burke TM Scheer FAJL Ronda JM Czeisler CA Wright KP Jr (2015) Sleep inertia, sleep homeostatic and circadian influences on higher-order cognitive functions. J Sleep Res 24:364-371. 25773686 10.1111/jsr.12291 PMC5124508

[bibr16-07487304221096945] Buxton OM Cain SW O’Connor SP Porter JH Duffy JF Wang W Czeisler CA Shea SA (2012) Adverse metabolic consequences in humans of prolonged sleep restriction combined with circadian disruption. Sci Trans Med 4:129ra43. 10.1126/scitranslmed.3003200 PMC3678519 22496545

[bibr17-07487304221096945] Buysse DJ Reynolds CF III Monk TH Berman SR Kupfer DJ (1989) The Pittsburgh Sleep Quality Index: a new instrument for psychiatric practice and research. Psychiatry Res 28:193-213. 2748771 10.1016/0165-1781(89)90047-4

[bibr18-07487304221096945] Cain SW Rimmer DW Duffy JF Czeisler CA (2007) Exercise distributed across day and night does not alter circadian period in humans. J Biol Rhythms 22:534-541. 18057328 10.1177/0748730407306884

[bibr19-07487304221096945] Cajochen C Münch M Kobialka S Kräuchi K Steiner R Oelhafen P Orgül S Wirz-Justice A (2005) High sensitivity of human melatonin, alertness, thermoregulation, and heart rate to short wavelength light. J Clin Endocrinol Metab 90:1311-1316. 15585546 10.1210/jc.2004-0957

[bibr20-07487304221096945] Cajochen C Wyatt JK Czeisler CA Dijk DJ (2002) Separation of circadian and wake duration-dependent modulation of EEG activation during wakefulness. Neuroscience 114:1047-1060. 12379258 10.1016/s0306-4522(02)00209-9

[bibr21-07487304221096945] Cajochen C Zeitzer JM Czeisler CA Dijk DJ (2000) Dose-response relationship for light intensity and ocular and electroencephalographic correlates of human alertness. Behav Brain Res 115:75-83. 10996410 10.1016/s0166-4328(00)00236-9

[bibr22-07487304221096945] Carskadon MA Labyak SE Acebo C Seifer R (1999) Intrinsic circadian period of adolescent humans measured in conditions of forced desynchrony. Neurosci Lett 260:129-132. 10025716 10.1016/s0304-3940(98)00971-9

[bibr23-07487304221096945] Curcio G Casagrande M Bertini M (2001) Sleepiness: evaluating and quantifying methods. Int J Psychophysiol 41:251-263. 11448507 10.1016/s0167-8760(01)00138-6

[bibr24-07487304221096945] Czeisler CA Duffy JF Shanahan TL Brown EN Mitchell JF Rimmer DW Ronda JM Silva EJ Allan JS Emens JS , et al. (1999) Stability, precision, and near-24-hour period of the human circadian pacemaker. Science 284:2177-2182. 10381883 10.1126/science.284.5423.2177

[bibr25-07487304221096945] Daan S Beersma DGM Borbély AA (1984) Timing of human sleep: recovery process gated by a circadian pacemaker. Am J Physiol 246:R161-R183. 6696142 10.1152/ajpregu.1984.246.2.R161

[bibr26-07487304221096945] Darwent D Ferguson SA Sargent C Paech GM Williams L Zhou X Matthews RW Dawson D Kennaway DJ Roach GD (2010) Contribution of core body temperature, prior wake time, and sleep stages to cognitive throughput performance during forced desynchrony. Chronobiol Int 27:898-910. 20636204 10.3109/07420528.2010.488621

[bibr27-07487304221096945] Dijk DJ Czeisler CA (1994) Paradoxical timing of the circadian rhythm of sleep propensity serves to consolidate sleep and wakefulness in humans. Neurosci Lett 16:663-668. 10.1016/0304-3940(94)90841-9 8190360

[bibr28-07487304221096945] Dijk DJ Edgar DM (1999) Circadian and homeostatic control of wakefulness and sleep. Lung Biol Health Dis 133:111-147.

[bibr29-07487304221096945] Dijk DJ Duffy JF Czeisler CA (1992) Circadian and sleep/wake dependent aspects of subjective alertness and cognitive performance. J Sleep Res 1:112-117. 10607036 10.1111/j.1365-2869.1992.tb00021.x

[bibr30-07487304221096945] Dijk DJ Duffy JF Kiel E Shanahan TL Czeisler CA (1999) Ageing and the circadian and homeostatic regulation of human sleep during forced desynchrony of rest, melatonin and temperature rhythms. J Physiol 516:611-627. 10087357 10.1111/j.1469-7793.1999.0611v.x PMC2269279

[bibr31-07487304221096945] Dinges DF Powell JW (1985) Microcomputer analyses of performance on a portable, simple visual RT task during sustained operations. Behav Res Meth Instrum Comput 17:652-655.

[bibr32-07487304221096945] Driver HS Dijk DJ Werth E Biedermann K Borbély AA (1996) Sleep and the sleep electroencephalogram across the menstrual cycle in young healthy women. J Clin Endocrinol Metab 81:728-735. 8636295 10.1210/jcem.81.2.8636295

[bibr33-07487304221096945] Duffy JF Cain SW Chang A-M Phillips AJK Münch M Gronfier C Wyatt JK Dijk DJ Wright KP Jr Czeisler CA (2011) Sex difference in the near-24-hour intrinsic period of the human circadian timing system. Proc Natl Acad Sci U S A 108:15602-15608. 21536890 10.1073/pnas.1010666108 PMC3176605

[bibr34-07487304221096945] Duffy JF Rimmer DW Czeisler CA (2001) Association of intrinsic circadian period with morningness-eveningness, usual wake time, and circadian phase. Behav Neurosci 115:895-899. 11508728 10.1037//0735-7044.115.4.895

[bibr35-07487304221096945] Ferguson SA Paech GM Sargent C Darwent D Kennaway DJ Roach GD (2012) The influence of circadian time and sleep dose on subjective fatigue ratings. Accid Anal Prev 45:50-54. 10.1016/j.aap.2011.09.026 22239932

[bibr36-07487304221096945] Figueiro MG Sahin L Wood BM Plitnick BA (2015) Light at night and measures of alertness and performance: implications for shift workers. Biol Res Nurs 18:90-100. 25697165 10.1177/1099800415572873

[bibr37-07487304221096945] Gabel V Kass M Joyce DS Spitschan M Zeitzer JM (2019) Auditory psychomotor vigilance testing in older and young adults: a revised threshold setting procedure. Sleep Breath 24:1021-1025. 10.1007/s11325-019-01859-7 31069648

[bibr38-07487304221096945] Gronfier C Wright KP Jr Kronauer RE Czeisler CA (2007) Entrainment of the human circadian pacemaker to longer-than-24-h days. Proc Natl Acad Sci U S A 104:9081-9086. 17502598 10.1073/pnas.0702835104 PMC1885631

[bibr39-07487304221096945] Hiddinga AE Beersma DGM Van den Hoofdakker RH (1997) Endogenous and exogenous components in the circadian variation of core body temperature in humans. J Sleep Res 6:156-163. 9358393 10.1046/j.1365-2869.1997.00047.x

[bibr40-07487304221096945] Hilton MF Umali MU Czeisler CA Wyatt JK Shea SA (2000) Endogenous circadian control of the human autonomic nervous system. Comput Cardiol 27:197-200. 14632012

[bibr41-07487304221096945] Hu K Ivanov PC Hilton MF Chen Z Ayers RT Stanley HE Shea SA (2004) Endogenous circadian rhythm in an index of cardiac vulnerability independent of changes in behavior. Proc Natl Acad Sci U S A 101:18223-18227. 15611476 10.1073/pnas.0408243101 PMC539796

[bibr42-07487304221096945] Hull JT Wright KP Jr Czeisler CA (2003) The influence of subjective alertness and motivation on human performance independent of circadian and homeostatic regulation. J Biol Rhythms 18:329-338. 12932085 10.1177/0748730403253584

[bibr43-07487304221096945] Ishihara S (1972) The series of plates designed as a tests for colour-blindness, in Tests for Colour-blindness. Tokyo: Kanehara Shuppan Co., Ltd.

[bibr44-07487304221096945] Ivanov PC Hu K Hilton MF Shea SA Stanley HE (2007) Endogenous circadian rhythm in human motor activity uncoupled from circadian influences on cardiac dynamics. Proc Natl Acad Sci U S A 104:20702-20707. 18093917 10.1073/pnas.0709957104 PMC2410066

[bibr45-07487304221096945] Johnson MP Duffy JF Dijk DJ Ronda JM Dyal CM Czeisler CA (1992) Short-term memory, alertness and performance: a reappraisal of their relationship to body temperature. J Sleep Res 1:24-29. 10607021 10.1111/j.1365-2869.1992.tb00004.x

[bibr46-07487304221096945] Jung CM Khalsa SBS Scheer FAJL Cajochen C Lockley SW Czeisler CA Wright KP Jr (2010) Acute effects of bright light exposure on cortisol levels. J Biol Rhythms 25:208-216. 20484692 10.1177/0748730410368413 PMC3686562

[bibr47-07487304221096945] Kendall A Lewy AJ Sack R (2001) Effects of aging on the intrinsic circadian period of totally blind humans. J Biol Rhythms 16:87-95. 11220783 10.1177/074873040101600110

[bibr48-07487304221096945] Klein DC Moore RY Reppert SM (1991) Suprachiasmatic nucleus: the mind’s clock. New York (NY): Oxford University Press.

[bibr49-07487304221096945] Kleitman N Kleitman E (1953) Effect of non-twenty-four-hour routines of living on oral temperature and heart rate. J Appl Physiol 6:283-291. 13108824 10.1152/jappl.1953.6.5.283

[bibr50-07487304221096945] Klerman EB Dijk DJ Kronauer RE Czeisler CA (1996) Simulations pacemaker: of light effects on the human circadian implications for assessment of intrinsic period. Am J Physiol 270:271-282. 10.1152/ajpregu.1996.270.1.R271 8769811

[bibr51-07487304221096945] Klerman EB Rimmer DW Dijk DJ Kronauer RE Rizzo JF Czeisler CA (1998) Nonphotic entrainment of the human circadian pacemaker. Am J Physiol 274:R991-R996. 9575961 10.1152/ajpregu.1998.274.4.r991

[bibr52-07487304221096945] Kline CE Durstine JL Davis JM Moore TA Devlin TM Youngstedt SD (2010) Circadian rhythms of psychomotor vigilance, mood, and sleepiness in the ultra-short sleep/wake protocol. Chronobiol Int 27:161-180. 20205564 10.3109/07420521003648604 PMC3248591

[bibr53-07487304221096945] Kolka MA Stephenson LA (1989) Control of sweating during the human menstrual cycle. Eur J Appl Physiol Occup Physiol 58:890-895. 2767071 10.1007/BF02332224

[bibr54-07487304221096945] Koorengevel KM Beersma DGM Den Boer JA van den Hoofdakker RH (2003) Mood regulation in seasonal affective disorder patients and healthy controls studied in forced desynchrony. Psychiatry Res 117:57-74. 12581821 10.1016/s0165-1781(02)00305-0

[bibr55-07487304221096945] Koorengevel KM Beersma DGM Gordijn MCM Den Boer JA van den Hoofdakker RH (2000) Body temperature and mood variations during forced desynchronization in winter depression: a preliminary report. Biol Psychiatry 47:355-358. 10686271 10.1016/s0006-3223(99)00225-5

[bibr56-07487304221096945] Kosmadopoulos A Sargent C Darwent D Zhou X Dawson D Roach GD (2014) The effects of a split sleep-wake schedule on neurobehavioural performance and predictions of performance under conditions of forced desynchrony. Chronobiol Int 31:1209-1217. 25222348 10.3109/07420528.2014.957763

[bibr57-07487304221096945] Kostoglou-Athanassiou I Treacher DF Wheeler MJ Forsling ML (1998) Bright light exposure and pituitary hormone secretion. Clin Endocrinol 48:73-79. 10.1046/j.1365-2265.1998.00355.x 9509071

[bibr58-07487304221096945] Kripke DF Youngstedt SD Elliott JA Tuunainen A Rex KM Hauger RL Marler MR (2005) Circadian phase in adults of contrasting ages. Chronobiol Int 22:695-709. 16147900 10.1080/07420520500180439

[bibr59-07487304221096945] Lavoie S Paquet J Selmaoui B Rufiange M Dumont M (2003) Vigilance levels during and after bright light exposure in the first half of the night. Chronobiol Int 20:1019-1038. 14680141 10.1081/cbi-120025534

[bibr60-07487304221096945] Lazar AS Lazar ZI Dijk DJ (2015) Circadian regulation of slow waves in human sleep: topographical aspects. Neuroimage 116:123-134. 25979664 10.1016/j.neuroimage.2015.05.012 PMC4503801

[bibr61-07487304221096945] Lee JH Wang W Silva EJ Chang A-M Scheuermaier KD Cain SW Duffy JF (2009) Neurobehavioral performance in young adults living on a 28-h day for 6 weeks. Sleep 32:905-913. 19639753 10.1093/sleep/32.7.905 PMC2706904

[bibr62-07487304221096945] Leproult R Colecchia EF Berardi AM Stickgold R Kosslyn SM Van Cauter E (2003) Individual differences in subjective and objective alertness during sleep deprivation are stable and unrelated. Am J Physiol 284:280-290. 10.1152/ajpregu.00197.2002 12529281

[bibr63-07487304221096945] Lok R Smolders KCHJ Beersma DGM de Kort YAW (2018a) Light, alertness, and alerting effects of white light: a literature overview. J Biol Rhythms 33:589-601. 30191746 10.1177/0748730418796443 PMC6236641

[bibr64-07487304221096945] Lok R Woelders T Gordijn MCM Hut RA Beersma DGM (2018b) White light during daytime does not improve alertness in well-rested individuals. J Biol Rhythms 33:637-648. 30191761 10.1177/0748730418796036 PMC6236585

[bibr65-07487304221096945] Mcintyre IM Norman TR Burrows GD Armstrong SM (1992) Melatonin, cortisol and prolactin response to acute nocturnal light exposure in healthy volunteers 17:243-248. 10.1016/0306-4530(92)90063-d 1438649

[bibr66-07487304221096945] Mifflin MD Jeor ST Hill LA Scott BJ Daugherty SA Koh YO (1990) A new predictive equation in healthy individuals for resting energy. American Journal of Clinical Nutrition 51:241-247. 2305711 10.1093/ajcn/51.2.241

[bibr67-07487304221096945] Monk TH Carrier J (1998) A parallelism between human body temperature and performance independent of the endogenous circadian pacemaker. J Biol Rhythms 13:113-122. 9554573 10.1177/074873098128999961

[bibr68-07487304221096945] Moore RY Eichler VB (1972) Loss of a circadian adrenal corticosterone rhythm following suprachiasmatic lesions in the rat. Brain Res Bull 72:54-56. 10.1016/0006-8993(72)90054-6 5047187

[bibr69-07487304221096945] Murphy PJ Campbell SS (1997) Nighttime drop in body temperature: a physiological trigger for sleep onset? Sleep 20:505-511. 9322266 10.1093/sleep/20.7.505

[bibr70-07487304221096945] O’Donnell D Silva EJ Münch M Ronda JM Wang W Duffy JF (2009) Comparison of subjective and objective assessments of sleep in healthy older subjects without sleep complaints. J Sleep Res 18:254-263. 19645969 10.1111/j.1365-2869.2008.00719.x PMC2975570

[bibr71-07487304221096945] Pardi D Buman M Black J Lammers GJ Zeitzer JM (2016) Eating decisions based on alertness levels after a single night of sleep manipulation: a randomized clinical trial. Sleep 40:1-8. 10.1093/sleep/zsw039 28364494

[bibr72-07487304221096945] Phipps-Nelson J Redman JR Dijk DJ Rajaratnam SMW (2003) Daytime exposure to bright Light, as compared to dim light, decreases sleepiness and improves psychomotor vigilance performance. Sleep 26:695-700. 14572122 10.1093/sleep/26.6.695

[bibr73-07487304221096945] Posner MI (2008) Measuring alertness. Ann N Y Acad Sci 1129:193-199. 18591480 10.1196/annals.1417.011

[bibr74-07487304221096945] Postnova S Lockley SW Robinson PA (2016) Sleep propensity under Forced Desynchrony in a model of arousal state dynamics. J Biol Rhythms 31:498-508. 27432116 10.1177/0748730416658806

[bibr75-07487304221096945] Postnova S Lockley SW Robinson PA (2018) Prediction of cognitive performance and subjective sleepiness using a model of arousal dynamics. J Biol Rhythms 33:203-218. 29671707 10.1177/0748730418758454

[bibr76-07487304221096945] Rahman SA Flynn-Evans EE Aeschbach D Brainard GC Czeisler CA Lockley SW (2014) Diurnal spectral sensitivity of the acute alerting effects of light. Sleep 37:271-281. 24501435 10.5665/sleep.3396 PMC3900613

[bibr77-07487304221096945] Ralph MR Foster RG Davis FC Menaker M (1990) Transplanted suprachiasmatic nucleus determines circadian period. Science 247:975-978. 2305266 10.1126/science.2305266

[bibr78-07487304221096945] Roenneberg T Wirz-Justice A Merrow M (2003) Life between clocks: daily temporal patterns of human chronotypes. J Biol Rhythms 18:80-90. 12568247 10.1177/0748730402239679

[bibr79-07487304221096945] Rollag MD Panke ES Trakulrungski W Trakulrungski C Reiter RJ (1980) Quantification of daily melatonin synthesis in the hamster pineal gland. Encorinology 106:231-236. 10.1210/endo-106-1-231 7349955

[bibr80-07487304221096945] Ruger M Gordijn MCM Beersma DGM de Vries B Daan S (2005) Time-of-day-dependent effects of bright light exposure on human psychophysiology: comparison of daytime and nighttime exposure. Am J Physiol 290:R1413-R1420. 10.1152/ajpregu.00121.2005 16373441

[bibr81-07487304221096945] Sargent C Darwent D Ferguson SA Kennaway DJ Roach GD (2012) Sleep restriction masks the influence of the circadian process on sleep propensity. Chronobiol Int 29:565-571. 22621352 10.3109/07420528.2012.675256

[bibr82-07487304221096945] Scheer FAJL Hilton MF Mantzoros CS Shea SA (2009) Adverse metabolic and cardiovascular consequences of circadian misalignment. Proc Natl Acad Sci U S A 106:4453-4458. 19255424 10.1073/pnas.0808180106 PMC2657421

[bibr83-07487304221096945] Scheer FAJL Hu K Evoniuk H Kelly EE Malhotra A Hilton MF Shea SA (2010) Impact of the human circadian system, exercise, and their interaction on cardiovascular function. Proc Natl Acad Sci U S A 107:20541-20546. 21059915 10.1073/pnas.1006749107 PMC2996667

[bibr84-07487304221096945] Scheer FAJL Michelson AD Frelinger AL Evoniuk H Kelly EE McCarthy M Doamekpor LA Barnard MR Shea SA (2011) The human endogenous circadian system causes greatest platelet activation during the biological morning independent of behaviors. PLoS ONE 6:e24549. 21931750 10.1371/journal.pone.0024549 PMC3169622

[bibr85-07487304221096945] Scheer FAJL van Doornen LJP Buijs RM (1999) Light and diurnal cycle affect human heart rate: possible role for the circadian pacemaker. J Biol Rhythms 14:202-212. 10452332 10.1177/074873099129000614

[bibr86-07487304221096945] Shea SA Hilton MF Hu K Scheer FAJL (2011) Existence of an endogenous circadian blood pressure rhythm in humans that peaks in the evening. Circ Res 108:980-984. 21474818 10.1161/CIRCRESAHA.110.233668 PMC3086568

[bibr87-07487304221096945] Silva EJ Wang W Ronda JM Wyatt JK Duffy JF (2010) Circadian and wake-dependent influences on subjective sleepiness, cognitive throughput, and reaction time performance in older and young adults. Sleep 33:481-490. 20394317 10.1093/sleep/33.4.481 PMC2849787

[bibr88-07487304221096945] Stephan FK Zucker I (1972) Circadian rhythms in drinking behavior and locomotor activity of rats are eliminated by hypothalamic lesions. Proc Natl Acad Sci U S A 691:583-586. 10.1073/pnas.69.6.1583 PMC426753 4556464

[bibr89-07487304221096945] Van Dongen HPA Maislin G Mullington JM Dinges DF (2003) The cumulative cost of additional wakefulness: dose-response effects on neurobehavioral functions and sleep physiology from chronic sleep restriction and total sleep deprivation. Sleep 26:117-126. 12683469 10.1093/sleep/26.2.117

[bibr90-07487304221096945] Waterhouse J Jones K Edwards B Harrison Y Nevill A Reilly T (2004) Lack of evidence for a marked endogenous component determining food intake in humans during forced desynchrony. Chronobiol Int 21:445-468. 15332449 10.1081/cbi-120038628

[bibr91-07487304221096945] Weber F (1967) Die Periodenlänge der Circadianen Laufperiodizität. Naturwissenschaften 54:122. 10.1007/BF00640591 5585839

[bibr92-07487304221096945] Wever RA (1977) The circadian system of man. Berlin (Germany): Springer.

[bibr93-07487304221096945] Woelders T Beersma DGM Gordijn MCM Hut RA Wams EJ (2017) Daily light exposure patterns reveal phase and period of the human circadian clock. J Biol Rhythms 32:174-286. 10.1177/0748730417696787 PMC5476188 28452285

[bibr94-07487304221096945] World Medical Association (2001) “World Medical Association Declaration of Helsinki. Ethical principles for medical research involving human subjects.” Bull World Health Organ 79(4):373–374. PMC2566407 11357217

[bibr95-07487304221096945] Wright KP Jr Hull JT Czeisler CA (2002) Relationship between alertness, performance, and body temperature in humans. Am J Physiol 283:R1370-R1377. 10.1152/ajpregu.00205.2002 12388468

[bibr96-07487304221096945] Wright KP Jr Hull JT Hughes RJ Ronda JM Czeisler CA (2006) Sleep and wakefulness out of phase with internal biological time impairs learning in humans. J Cogn Neurosci 18:508-521. 16768357 10.1162/jocn.2006.18.4.508

[bibr97-07487304221096945] Wright KP Jr Gronfier C Duffy JF Czeisler CA (2005) Intrinsic period and light intensity determine the phase relationship between melatonin and sleep in humans. J Biol Rhythms 20:168-177. 15834113 10.1177/0748730404274265 PMC2714089

[bibr98-07487304221096945] Wu LJ Acebo C Seifer R Carskadon MA (2015) Sleepiness and cognitive performance among younger and older adolescents across a 28-hour forced desynchrony protocol. Sleep 38:1965-1972. 26194564 10.5665/sleep.5250 PMC4667372

[bibr99-07487304221096945] Wyatt JK Cajochen C Ritz-De Cecco A Czeisler CA Dijk DJ (2004) Low-dose repeated caffeine administration for circadian-phase-dependent performance degradation during extended wakefulness. Sleep 27:374-381. 15164887 10.1093/sleep/27.3.374

[bibr100-07487304221096945] Wyatt JK Dijk DJ Ritz-de Cecco A Ronda JM Czeisler CA (2006) Sleep-facilitating effect of exogenous melatonin in healthy young men and women is circadian-phase dependent. Sleep 29:609-618. 16774150 10.1093/sleep/29.5.609

[bibr101-07487304221096945] Wyatt JK Ritz-De Cecco A Czeisler CA Dijk DJ (1999) Circadian temperature and melatonin rhythms, sleep, and neurobehavioral function in humans living on a 20-h day. Am J Physiol 277:1152-1163. 10.1152/ajpregu.1999.277.4.r1152 10516257

[bibr102-07487304221096945] Zhou X Ferguson SA Matthews RW Sargent C Darwent D Kennaway DJ Roach GD (2011a) Dynamics of neurobehavioral performance variability under forced desynchrony: evidence of state instability. Sleep 34:57-63. 21203373 10.1093/sleep/34.1.57 PMC3001796

[bibr103-07487304221096945] Zhou X Ferguson SA Matthews RW Sargent C Darwent D Kennaway DJ Roach GD (2011b) Sleep, wake and phase dependent changes in neurobehavioral function under forced desynchrony. Sleep 34:931-941. 21731143 10.5665/SLEEP.1130 PMC3119835

[bibr104-07487304221096945] Zhou X Ferguson SA Matthews RW Sargent C Darwent D Kennaway DJ Roach GD (2012) Mismatch between subjective alertness and objective performance under sleep restriction is greatest during the biological night. J Sleep Res 21:40-49. 21564364 10.1111/j.1365-2869.2011.00924.x

[bibr105-07487304221096945] Zitting KM Vujovic N Yuan RK Isherwood CM Medina JE Wang W Buxton OM Williams JS Czeisler CA Duffy JF (2018) Human resting energy expenditure varies with circadian phase. Curr Biol 28:3685-3690.e3. 10.1016/j.cub.2018.10.005 PMC6300153 30416064

