# Supplementary material for: Bright Light Increases Alertness and Not Cortisol in Healthy Men: A Forced Desynchrony Study Under Dim and Bright Light (I)
Source: J Biol Rhythms. 2022 Jun 10;37(4):403–16. doi: 10.1177/07487304221096945 (PMC9326799; doi:10.1177/07487304221096945)
Supplement: sj-docx-1-jbr-10.1177_07487304221096945 – Supplemental material for Bright Light Increases Alertness and Not Cortisol in Healthy Men: A Forced Desynchrony Study Under Dim and Bright Light (I) [file sj-docx-1-jbr-10.1177_07487304221096945.docx]

**Title:** Bright light increases alertness and not cortisol in healthy men: a forced desynchrony study under dim and bright light (I)

**Running title:** Bright light increases alertness.

**Authors:** R. Lok^1,2,3,^*, T. Woelders^1,3^, M.J. van Koningsveld^1^, K. Oberman^1^, S.G. Fuhler^1^, D.G.M. Beersma^1^, R.A. Hut^1^

**Contact Information: ^1^**University of Groningen, Chronobiology unit, Groningen Institute for Evolutionary Life Sciences, PO box 11103, 9700CC, Groningen, the Netherlands.

**^2^**University of Groningen, Campus Fryslân, Wirdumerdijk 34, 8911 CE, Leeuwarden, the Netherlands.

^3^ Should be considered as joint first author.

^*^ To whom all correspondence should be addressed: Renske Lok, University of Groningen, Chronobiology Unit, Present address: Department of Psychiatry and Behavioral Sciences, Stanford University, 401 Quarry Road, Palo Alto, CA, 94305, [rlok@stanford.edu](mailto:rlok@stanford.edu)

**Supplemental information**


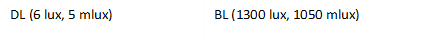

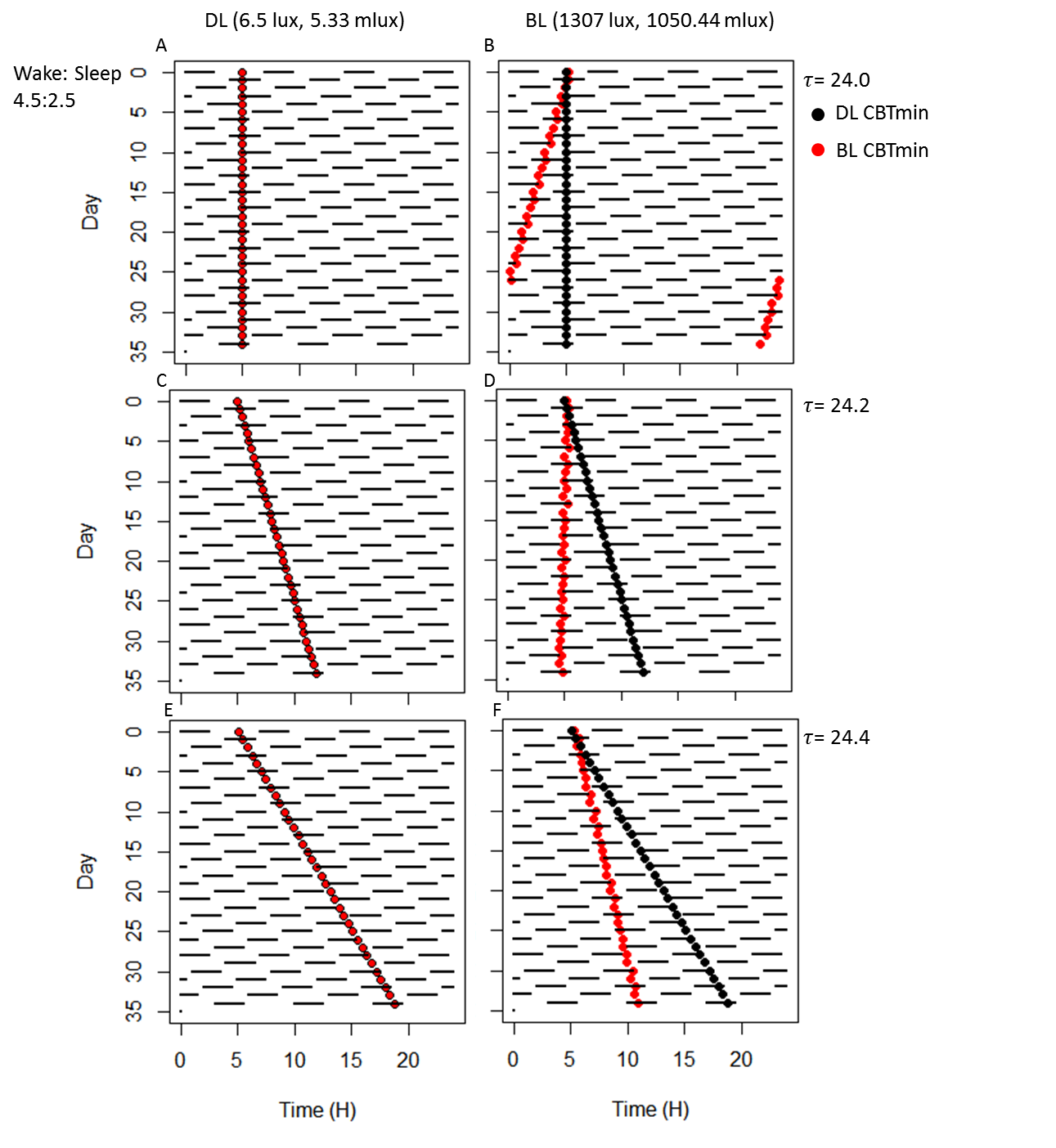


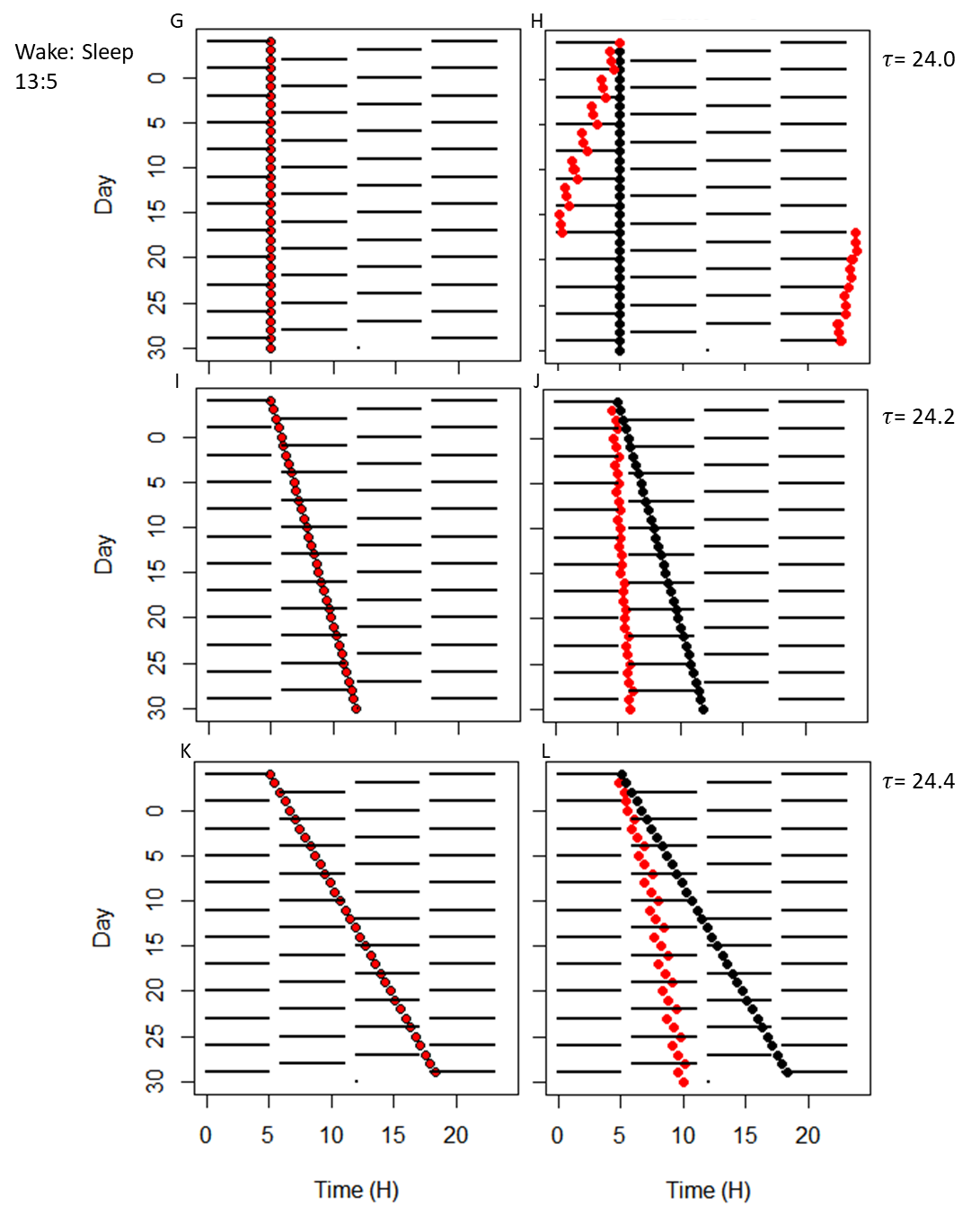


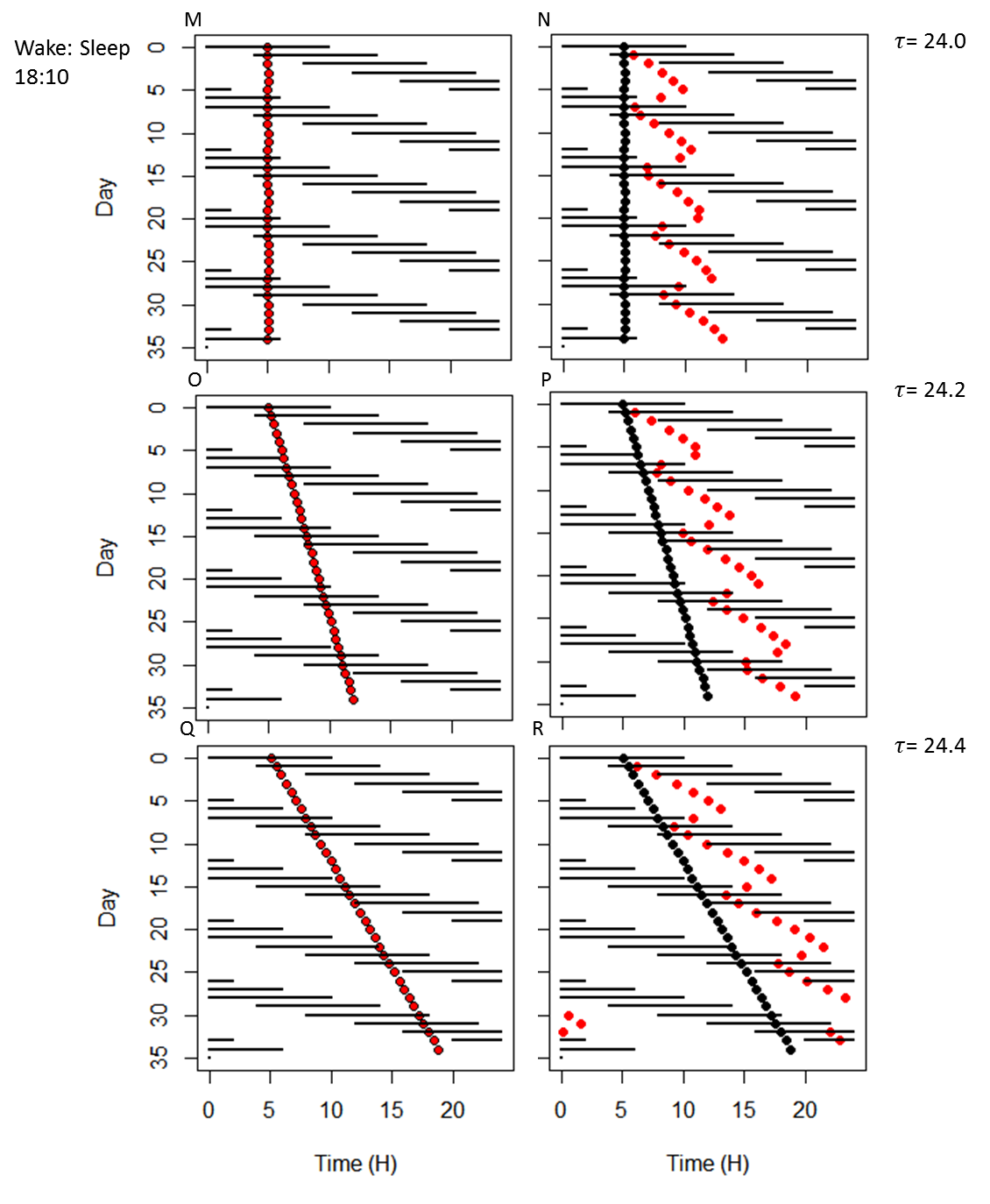


**Figure S1. An adjusted version of the Kronauer model was used to model clock phase changes under dim light (DL; 5 melanopic lux, 6 lux) and bright light (BL; 1050 melanopic lux, 1300 lux) conditions.** Three different wake:sleep amounts were modelled, 4.5:2.5 (A:F), 13:5 (G:L), and 18:10 (M:R), for three different internal period lengths (𝜏) of 24.0, 24.2 or 24.4 hours. Every horizontal bar represents a day, while every dot indicates CBTmin. Although there is uniform phase progression under all wake:sleep amounts in DL, simulations indicate lower amounts of non-uniform phase progression under shorter wake:sleep cycles in BL.

**
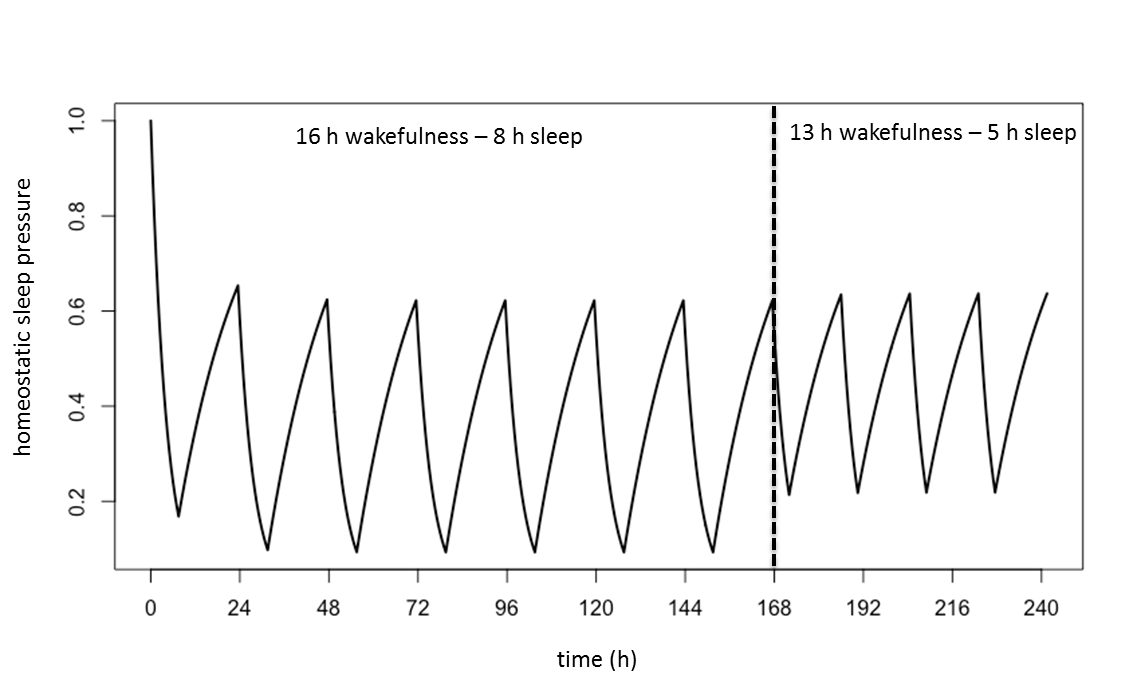
Figure S2. Simulations of sleep pressure build-up under normal, rested conditions (16h wakefulness – 8h sleep) versus sleep pressure build-up during 13h wakefulness – 5h sleep conditions.** Simulations indicate that under 13h wakefulness and 5h sleep, systematic fluctuations of sleep pressure over the sleep-wake schedule does not change over the course of the FD protocol. Compared to normal sleep timing, sleep pressure in forced desynchrony at awakening is relatively high, while it is about normal at the end of the wake interval.

**Table S1: Photometric properties of dim- and bright light according to CIE (in α-opic EDI lux)**(CIE, 2018) **and Lucas toolbox (in α-opic lux)** (Lucas et al., 2014)**.**

|  | Dim light | Bright light | Unit |
| --- | --- | --- | --- |
| Peak irradiance | 545 | | nm |
| Illuminance | 6.50 | 1307.00 | lux |
| S-cone-opic | 6.63 | 1350.17 | α-opic EDI lux |
|  | 7.01 | 1433.41 | α-opic lux |
| M-cone-opic | 6.03 | 1196.58 | α-opic EDI lux |
|  | 6.28 | 1247.15 | α-opic lux |
| L-cone-opic | 6.61 | 1319.13 | α-opic EDI lux |
|  | 6.50 | 1397.23 | α-opic lux |
| Melanopic | 5.33 | 1050.44 | α-opic EDI lux |
|  | 5.88 | 1159.27 | α-opic lux |
| Rhodopic | 5.46 | 1076.10 | α-opic EDI lux |
|  | 6.03 | 1189.46 | α-opic lux |


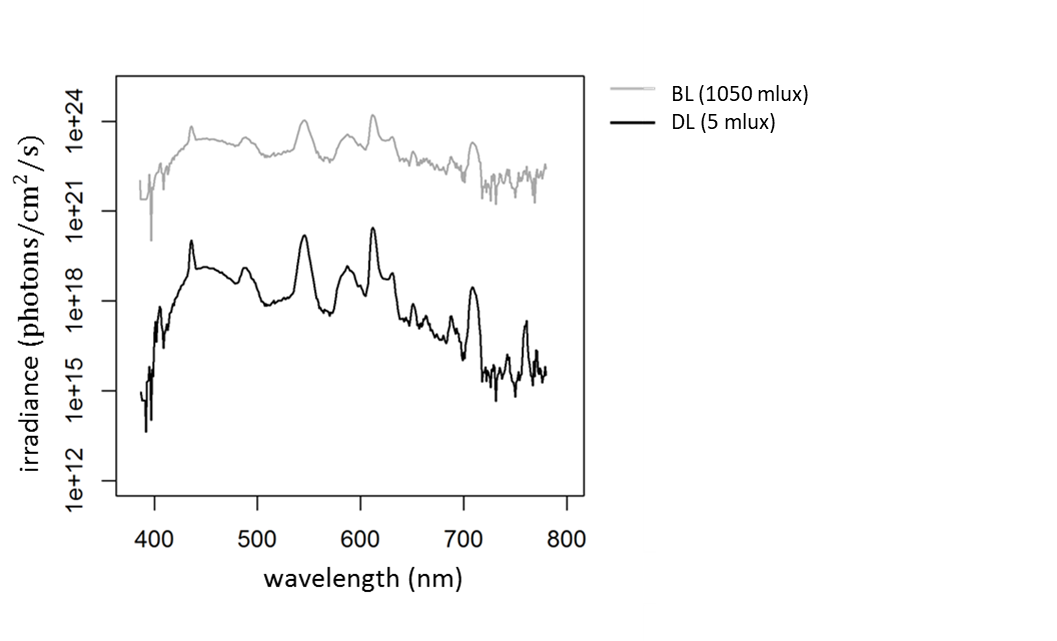


**Figure S3:** **Spectral composition of dim- (black) and bright light (grey).** Illuminance was measured on the vertical plane at the level of the eye. Light was generated with ceiling-mounted Philips fluorescent light tubes.


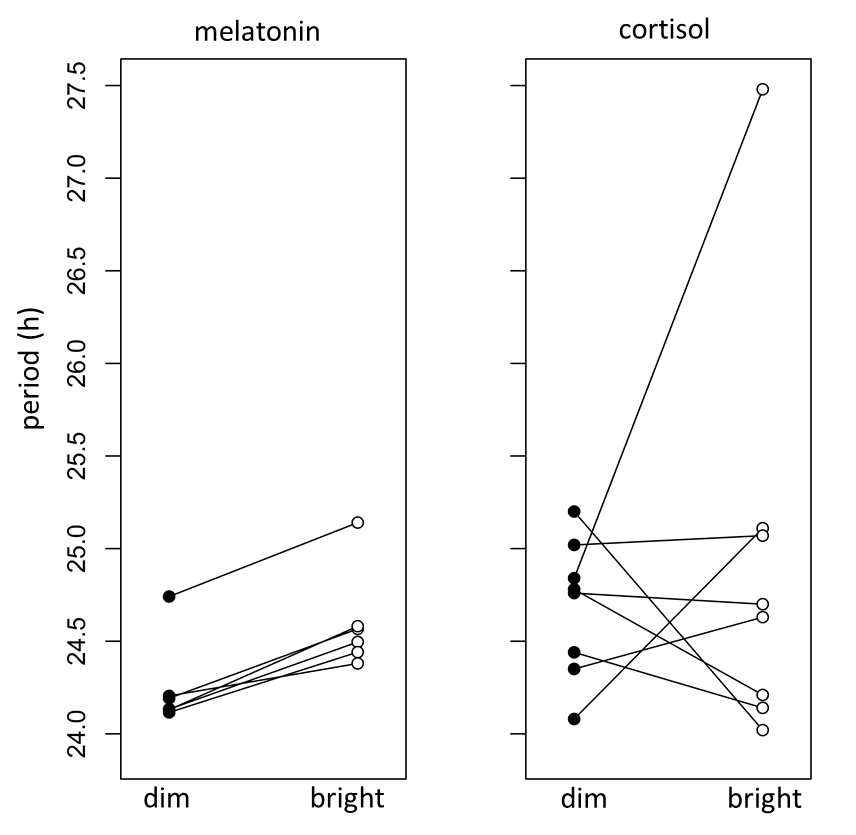


**Figure S4: Internal period (**$\boldsymbol{\tau}$**) in DL and BL based on melatonin and cortisol concentrations**. Black and white dots represent individual data points collected in DL and BL respectively. Average increase of circadian period under BL versus DL was 21 min.


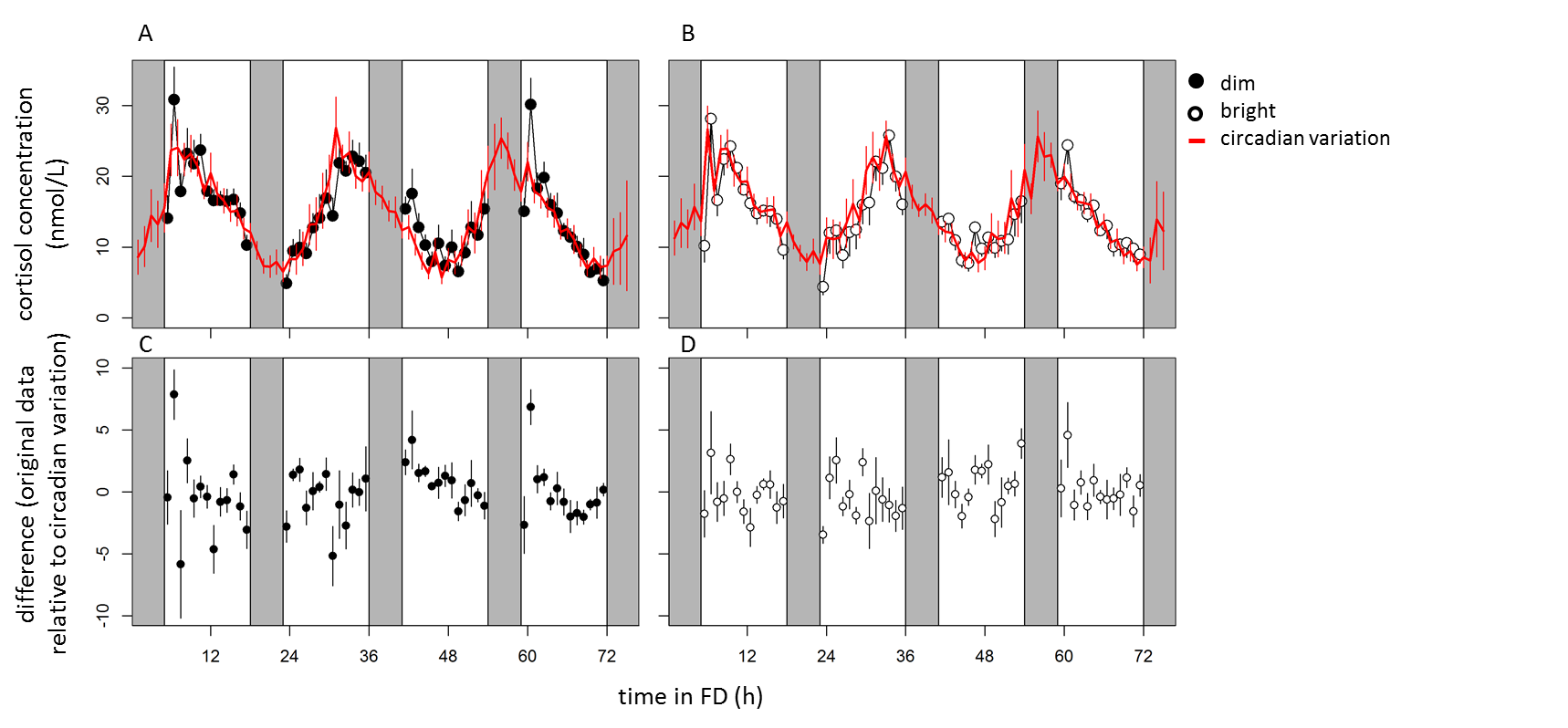
**Figure S5:** **Stable free run with uniform phase progression in cortisol levels.** Original cortisol concentrations collected during dim (A) and bright light exposure (B) demonstrating their respective circadian variations. After subtracting the calculated circadian variation from the data, the residuals were plotted against time in FD for both DL (C) and BL (D). The residual data (C-D) did not depict a significant circadian not 72-h modulation (p>0.05). The residual variation was 1.61 (DL) and 1.83% (BL) of total variation in the raw data.

**Performance: PVT.** 10% slowest and fastest PVT reaction times indicated a significant effect of light exposure independent of time awake (Fig S6B, S6E Table S2). The number omission errors were significantly impacted by wakefulness duration, but not by light exposure (Fig S6H). Number of lapses, according to the classical definition (in which a lapse is a response time over 500 ms) indicated significant light effects independent of time awake. Circadian clock time did not significantly affect 10% slowest or fastest reaction times, nor lapses, although bright light exposure did significantly improve these reaction times (Fig S6C, S6F, Table S2). Errors of omissions were not significantly affected by circadian clock phase or light exposure (Fig S6I, Table S2). The number of lapses were relatively high compared to literature, which is due to some subjects that were consistently slower over the course of the protocol.


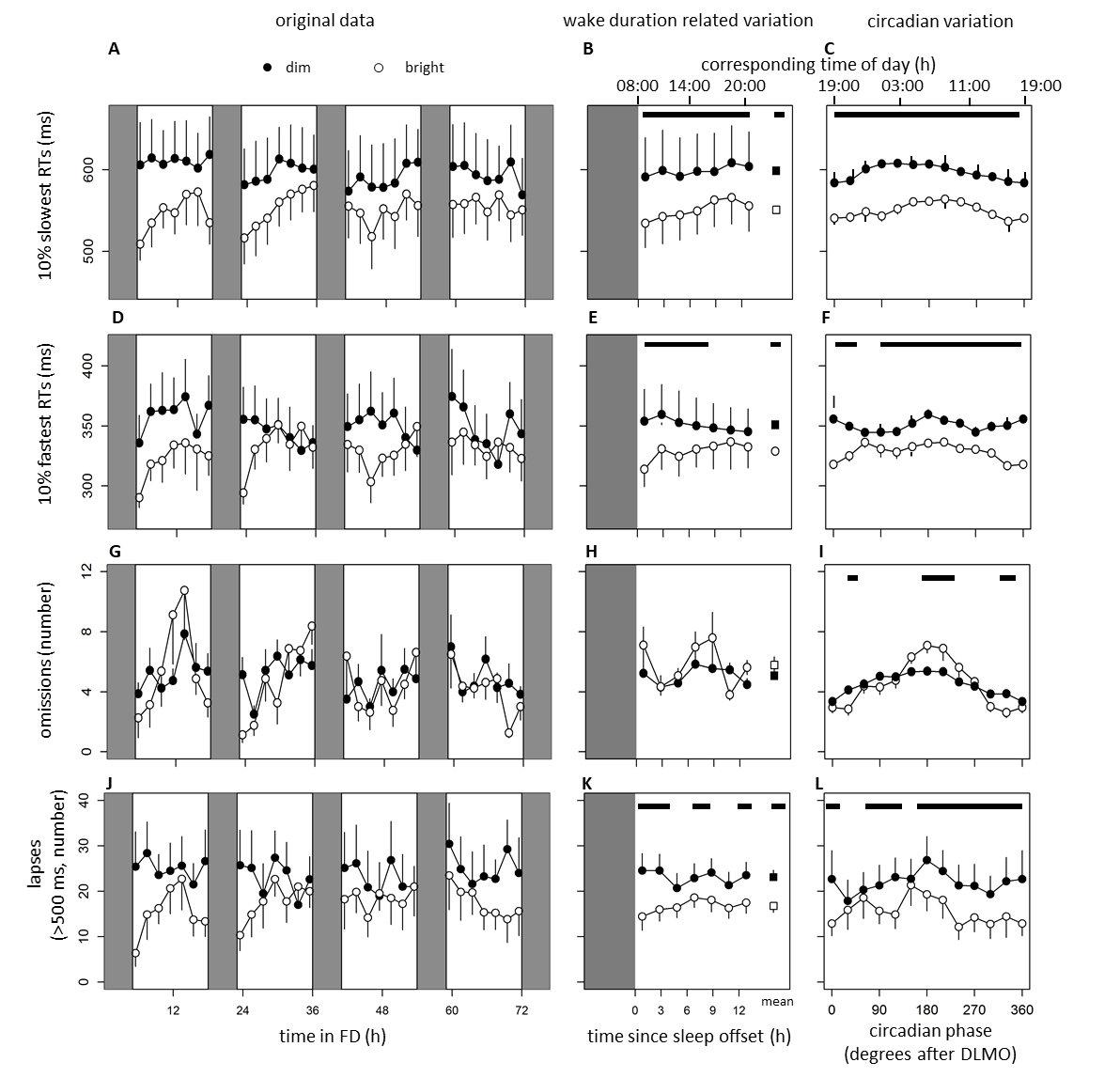


**Figure S6: Data of 10% slowest (top panels), and fastest reaction times, number of omissions (middle panels) and number of lapses (500 ms cutoff, bottom panels) on the PVT.** Time course of 10% slowest (A), and fastest reaction times (D), number of omissions (G) and number of lapses (according to the classical definition of >500 ms, J) on the PVT task during the FD protocol. Data replotted as time since sleep offset (B,E, H, K) and circadian phase in degrees after DLMO (C, F, I, L), for 10% slowest, and fastest reaction times, number of omission and number of lapses respectively. Data represent mean ± standard error of the mean, with 7 subjects per group. Black dots indicate data collected in dim light, white dots represent data collected in bright light and black and white squares represent averages over all data points under DL and BL respectively. Shaded areas represent scheduled sleep (at 0 lux). Significant differences between light conditions (p<0.05) are indicated by horizontal black bars.

**Performance: GNG.** 10% slowest reaction times, number of omissions and commissions were not significantly impacted by time awake (Fig S7B, S7H, S7N, Table S2), while 10% fastest RTs and anticipation errors did significantly change with increasing wakefulness duration (Fig S7E, S7K, Table S2). Light significantly decreased 10% slowest and fastest reaction times, as well as number of anticipation errors (Fig S7B, S7K), while errors of omission and commission were unaffected (Fig S7H, S7N, Table 2). There were no significant effects of internal clock time on none of the GNG measures (Fig S7C, S7F, S7I, S7L, S7O), although bright light did decrease 10% slowest and fastest reaction times (Fig S7C, Fig S7F).


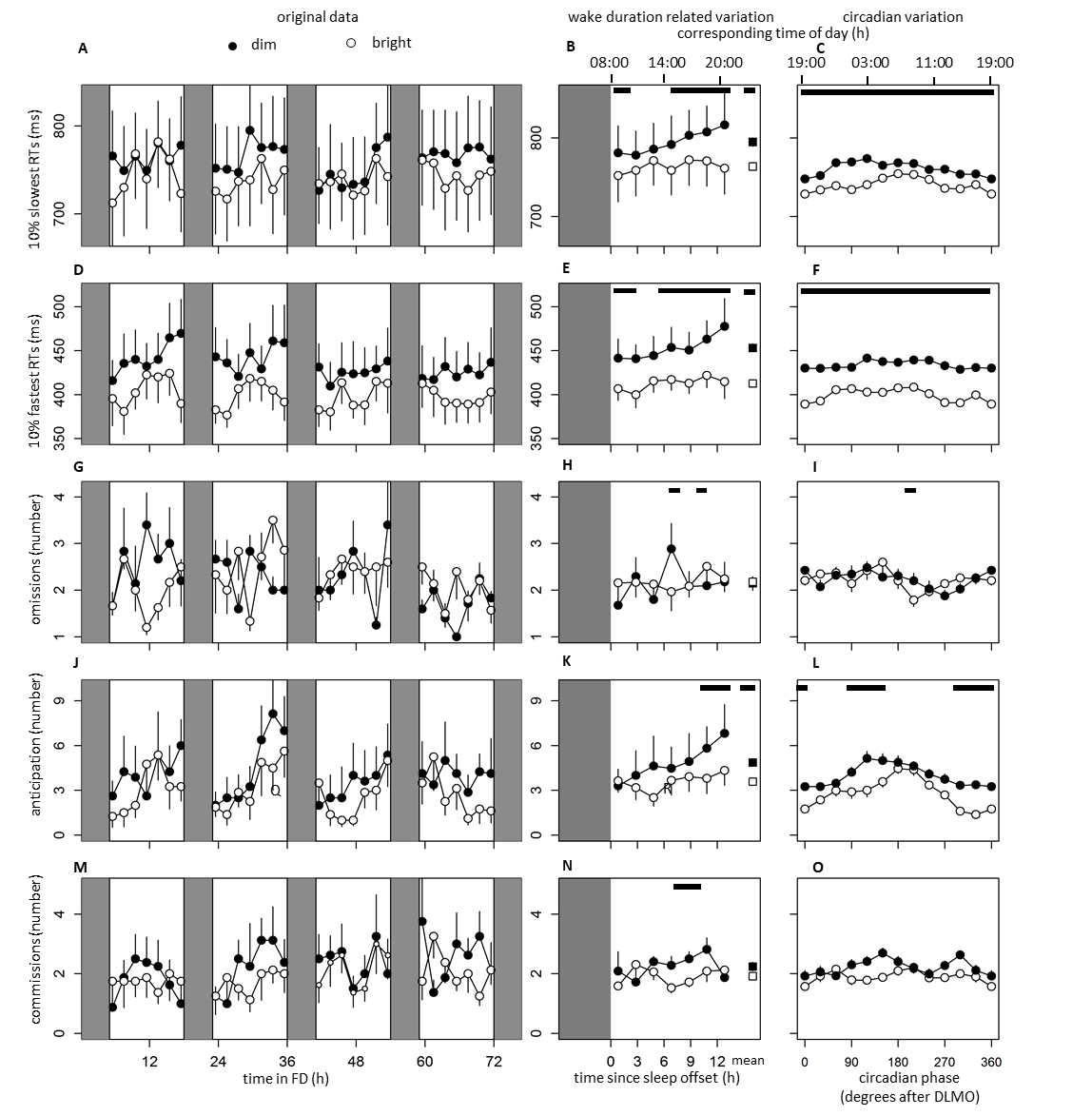


**Figure S7: Data of 10% slowest reaction times (top panels), 10% fastest reaction times, number of omissions and anticipation errors (middle panels) and number of commissions (bottom panels) on the GNG.** Time course of 10%slowest (A) and fastest reaction times (D), as well as errors of omissions (G), anticipation (J) and commission (M) on the GNG task during the FD protocol. Data replotted as time since sleep offset (B,E, JH, K, N) and circadian phase in degrees after DLMO (C, F, I, L, O), for 10%slowest and fastest reaction times, errors of omission, anticipation and commission respectively. Data represent mean ± standard error of the mean, with 7 subjects per group. Black dots indicate data collected in dim light, white dots represent data collected in bright light and black and white squares represent averages over all data points under DL and BL respectively. Shaded areas represent scheduled sleep (at 0 lux). Significant differences between light conditions (p<0.05) are indicated by horizontal black bars.

**Table S2: Summary of statistics of sleep-wake related variation (process S), circadian variation (process C), interaction between process S and C, and additive effects of bright light exposure.** Values from linear mixed models on 10% slowest and fastest reaction times, errors of omissions, commissions and anticipation.

|  |  | **Wake duration related variation**  **(process S)** | | **Circadian variation**  **(process C)** | | **Interaction**  **(process S x C)** | | **Additive effect of bright light** | |
| --- | --- | --- | --- | --- | --- | --- | --- | --- | --- |
| **PVT** | **10% slowest** | *F_(6, 392),_ p* | 1.42,  >0.05 | *F_(5, 392),_*  *p* | 0.00,  >0.05 | *F_(30, 392),_*  *p* | 0.00,  >0.05 | *F_(1, 392),_*  *p* | **112.32,**  **<0.00001** |
|  | **10% fastest** | *F_(6, 392),_ p* | 1.61,  p>0.05 | *F_(5, 392),_*  *p* | 0.00,  >0.05 | *F_(30, 392),_*  *p* | 0.00,  >0.05 | *F_(1, 392),_*  *p* | **86.35,**  **<0.00001** |
|  | **Omissions** | *F_(6, 392),_ p* | **8.51,**  **<0.0001** | *F_(5, 392),_*  *p* | 0.00,  >0.05 | *F_(30, 392),_*  *p* | 0.00,  >0.05 | *F_(1, 392),_*  *p* | 0.43,  >0.05 |
|  | **Lapses** | *F_(6, 392),_ p* | 0.22,  >0.05 | *F_(5, 392),_*  *p* | 0.46,  >0.05 | *F_(30, 392),_*  *p* | 1.17,  >0.05 | *F_(1, 392),_*  *p* | **105.81,**  **<0.00001** |
| **GNG** | **10% slowest** | *F_(6, 392),_ p* | 1.61,  >0.05 | *F_(5, 392),_*  *p* | 0.00,  >0.05 | *F_(30, 392),_*  *p* | 0.00,  >0.05 | *F_(1, 392),_*  *p* | **63.45,**  **<0.00001** |
|  | **10% fastest** | *F_(6, 392),_ p* | **2.13,**  **<0.05** | *F_(5, 392),_*  *p* | 0.00,  >0.05 | *F_(30, 392),_*  *p* | 0.00,  >0.05 | *F_(1, 392),_*  *p* | **38.31,**  **<0.00001** |
|  | **Omissions** | *F_(6, 392),_ p* | 0.00,  >0.05 | *F_(5, 392),_*  *p* | 0.00,  >0.05 | *F_(30, 392),_*  *p* | 0.00,  >0.05 | *F_(1, 392),_*  *p* | 0.00,  >0.05 |
|  | **Anticipation** | *F_(6, 392),_ p* | **17.59,**  **<0.001** | *F_(5, 392),_*  *p* | 0.00,  >.05 | *F_(30, 392),_*  *p* | 0.00,  >0.05 | *F_(1, 392),_*  *p* | **21.14,**  **<0.0001** |
|  | **Commissions** | *F_(6, 392),_ p* | 0.60,  >0.05 | *F_(5, 392),_*  *p* | 0.00,  >0.05 | *F_(30, 392),_*  *p* | 0.00,  >0.05 | *F_(1, 392),_*  *p* | 0.00,  >0.05 |

**EEG based indices of alertness.** Wake EEG analysis was measured in the electrode placed at frontal, central and occipital locations (Fig S8, S9, S10). Data revealed a significant effect of light exposure on alpha power with eyes open independent of time awake (Fig S8B, S9B, S10B, Table S3). Circadian clock time did not significantly affect alpha power density, although this was significantly decreased by BL exposure (Fig S8C, S9C, S10C Table S3).

Alpha band EEG activity when eyes were closed was independent of time awake, but significantly decreased by BL exposure (Fig S8E, S9E, S10E, Table S3). There were no significant effects of circadian clock time in all electrode placements (Fig S8F, S9F, S10F, Table S3), but significant decreases due to BL exposure were only found in the central electrode (Fig S9F).

Independent of wakefulness duration effects, there was a significant effect of light exposure on theta power with eyes of in the frontal electrode (Fig S8H, Table S3) which was not detected in other electrode placements (Fig S9H, S10H, Table 3). There were no significant effects of circadian clock time (Fig S8I, S9I, S10I), but there was a significant bright light induced decrease in theta power with eyes open measured in the frontal electrode (Fig S8I, Table S3). No additive light effects were found in the other electrode placements (Fig S9I, S10I, Table S3).

When eyes were closed, theta power was not significantly affected by time awake (Fig S8K, S9K, S10K). Light significantly decreased theta power measured in the frontal electrode (Fig S8K, Table S3) but not in other electrode placements (Fig S9K, S10K, Table 3). Theta power measured when eyes were closed was not significantly affected by circadian clock time, but there was a significant bright light induced decrease measured in the frontal electrode (Fig S8L, Table 3). No additive light effects were found in other electrode locations (Fig S9L, S10L, Table 3).

There were significant differences between derivations (Fig S8, S9, S10), which is not unexpected, as this has been described in other papers ^2^


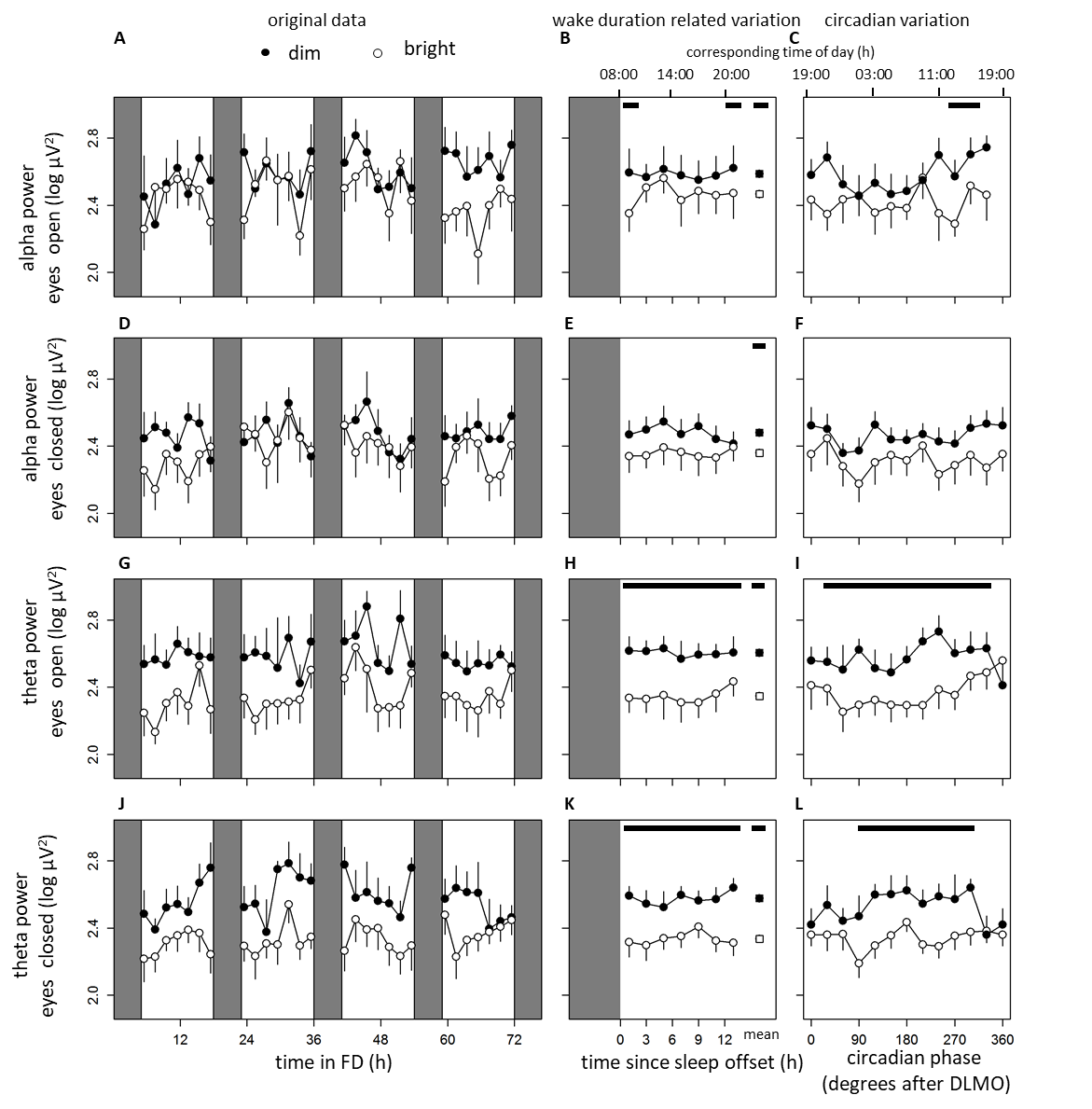


**Figure S8: Data of alpha power with eyes open and closed (top panels) and theta power with eyes open and closed (bottom panels) measured in the frontal electrodes.** Time course of alpha power with eyes open (A), and closed (E), as well as theta power with eyes open (G) and closed (J) during the FD protocol. Data replotted as time since sleep offset (B, E, H, K) and circadian phase in degrees after DLMO (C, F, I, L), for alpha power with eyes open and closed, as well as theta power with eyes open and closed respectively. Data represent mean ± standard error of the mean, with 7 subjects per group. Black dots indicate data collected in dim light, white dots represent data collected in bright light and black and white squares represent averages over all data points under DL and BL respectively. Shaded areas represent scheduled sleep (at 0 lux). Significant differences between light conditions (p<0.05) are indicated by horizontal black bars.


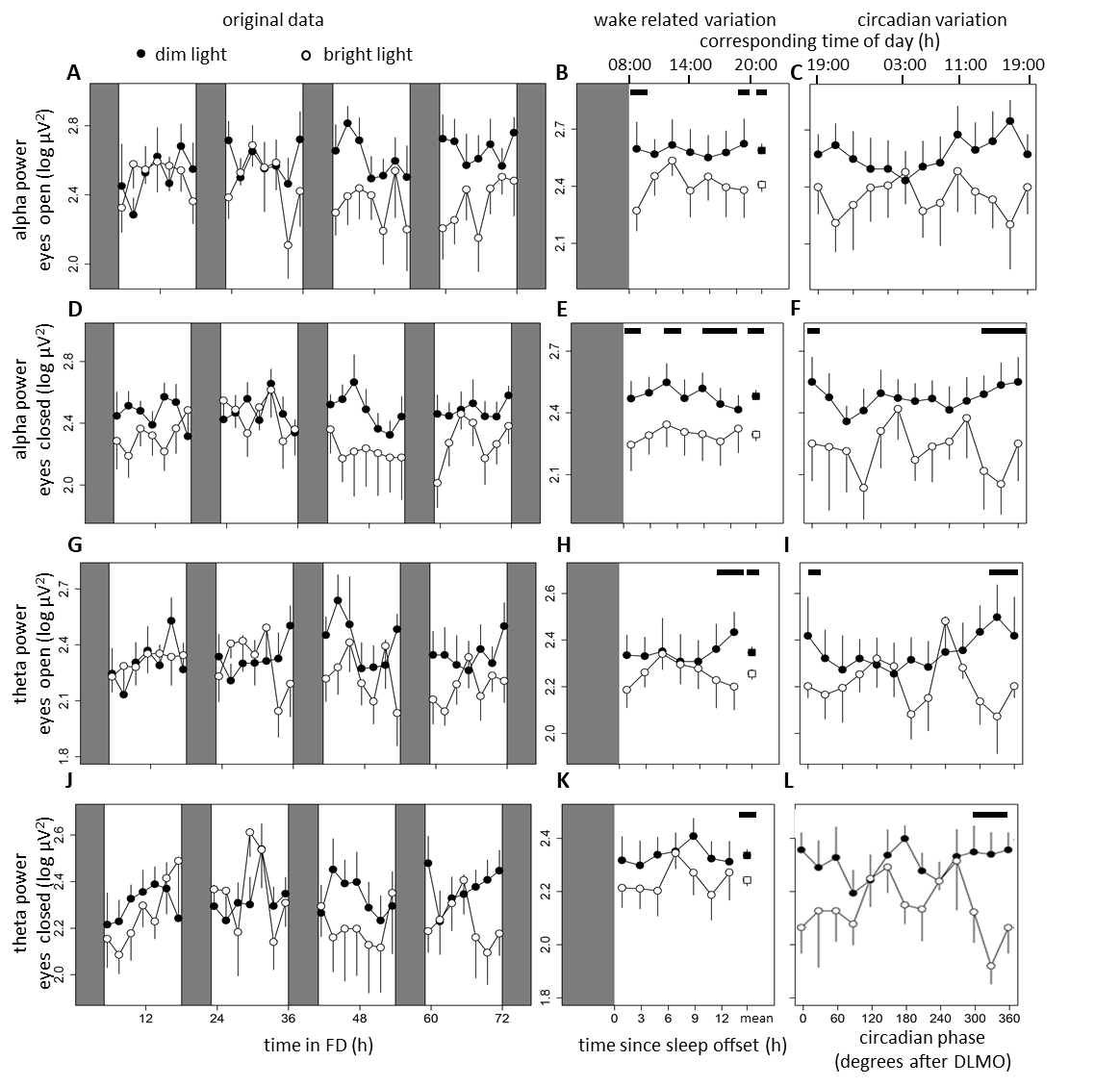


**Figure S9: Data of alpha power with eyes open and closed (top panels) and theta power with eyes open and closed (bottom panels) measured in the central electrode.** Time course of alpha power with eyes open (A), and closed (E), as well as theta power with eyes open (G) and closed (J) during the FD protocol. Data replotted as time since sleep offset (B, E, H, K) and circadian phase in degrees after DLMO (C, F, I, L), for alpha power with eyes open and closed, as well as theta power with eyes open and closed respectively. Data represent mean ± standard error of the mean, with 7 subjects per group. Black dots indicate data collected in dim light, white dots represent data collected in bright light and black and white squares represent averages over all data points under DL and BL respectively. Shaded areas represent scheduled sleep (at 0 lux). Significant differences between light conditions (p<0.05) are indicated by horizontal black bars.


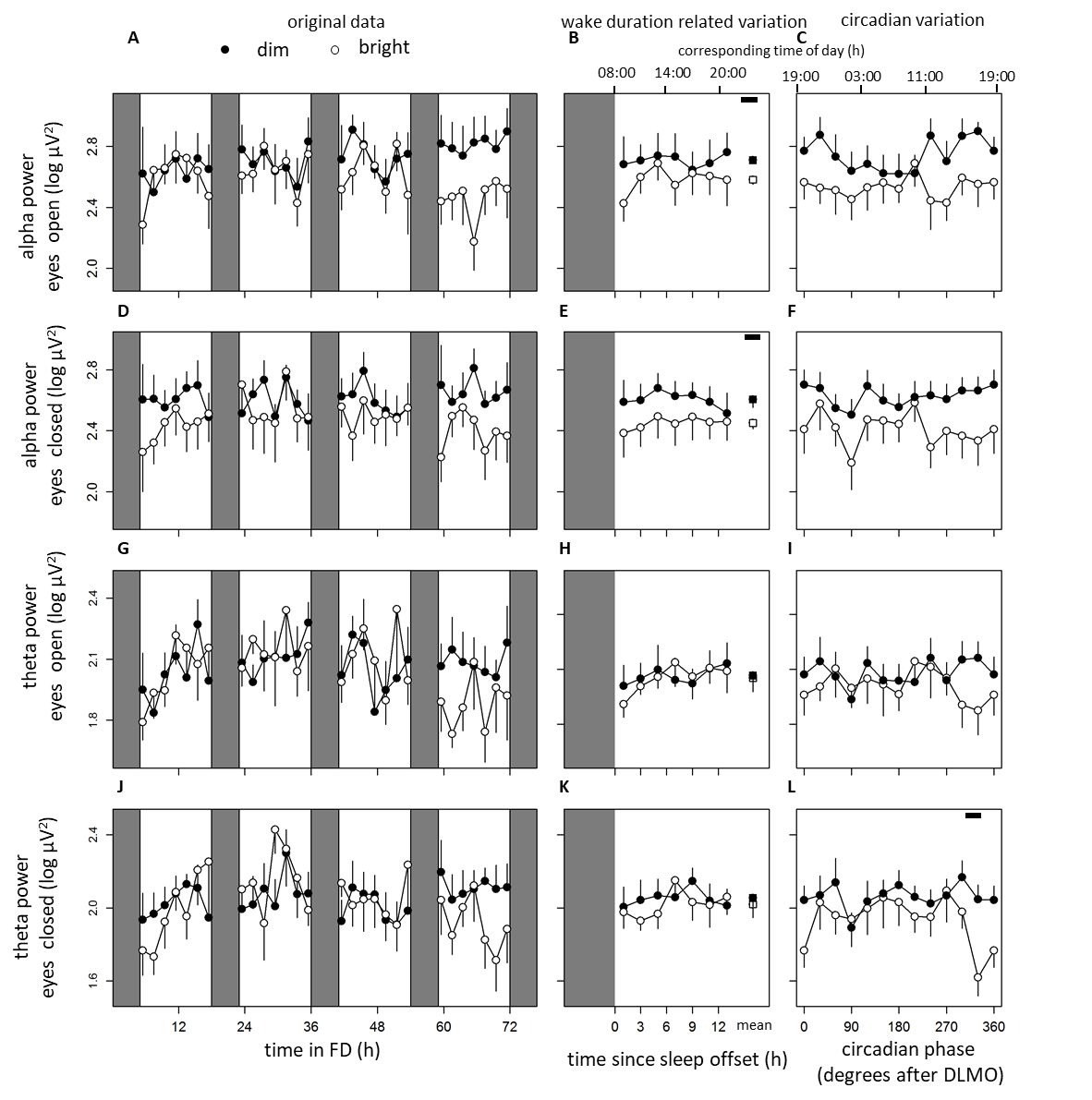


**Figure S10: Data of alpha power with eyes open and closed (top panels) and theta power with eyes open and closed (bottom panels) measured in the occipital electrodes.** Time course of alpha power with eyes open (A), and closed (E), as well as theta power with eyes open (G) and closed (J) during the FD protocol. Data replotted as time since sleep offset (B, E, H, K) and circadian phase in degrees after DLMO (C, F, I, L), for alpha power with eyes open and closed, as well as theta power with eyes open and closed respectively. Data represent mean ± standard error of the mean, with 7 subjects per group. Black dots indicate data collected in dim light, white dots represent data collected in bright light and black and white squares represent averages over all data points under DL and BL respectively. Shaded areas represent scheduled sleep (at 0 lux). Significant differences between light conditions (p<0.05) are indicated by horizontal black bars.

**Table S3: Summary of statistics of wake duration related variation (process S), circadian variation (process C), interaction between process S and C, and additive effects of bright light exposure.** Values from linear mixed models on EEG based indices of alertness, such as alpha activity with eyes open and closed, and theta activity with eyes open closed in frontal and occipital electrodes.

|  |  | **Wake duration related variation**  **(process S)** | | **Circadian variation**  **(process C)** | | **Interaction**  **(process C x S)** | | **Additive effect of bright light** | |
| --- | --- | --- | --- | --- | --- | --- | --- | --- | --- |
| **Frontal** | **Alpha eyes open** | *F_(6, 392),_ p* | 0.52,  >0.05 | *F_(5, 392),_*  *p* | 1.77,  >0.05 | *F_(30, 392),_ p* | **1.61,**  **<0.05** | *F_(1, 392),_*  *p* | **8.24,**  **<0.01** |
|  | **Alpha eyes closed** | *F_(6, 392),_ p* | 0.42,  >0.05 | *F_(5, 392),_*  *p* | 1.78,  >0.05 | *F_(30, 392),_ p* | 2.76,  <0.0001 | *F_(1, 392),_*  *p* | **11.17,**  **<0.001** |
|  | **Theta eyes open** | *F_(6, 392),_ p* | 0.54,  >0.05 | *F_(5, 392),_*  *p* | 1.99,  >0.05 | *F_(30, 392),_ p* | 0.92,  >0.05 | *F_(1, 392),_*  *p* | **55.48,**  **<0.00001** |
|  | **Theta eyes closed** | *F_(6, 392),_ p* | 0.46,  >0.05 | *F_(5, 392),_*  *p* | 1.46,  >0.05 | *F_(30, 392),_ p* | 0.81,  >0.05 | *F_(1, 392),_*  *p* | **56.39,**  **<0.00001** |
| **Central** | **Alpha eyes open** | *F_(6, 392),_ p* | 0.76,  >0.05 | *F_(5, 392),_*  *p* | 1.445,  >0.05 | *F_(30, 392),_ p* | 0.64,  >0.05 | *F_(1, 392),_*  *p* | **20.71,**  **<0.00001** |
|  | **Alpha eyes closed** | *F_(6, 392),_ p* | 0.57,  >0.05 | *F_(5, 392),_*  *p* | 1.10,  >0.05 | *F_(30, 392),_ p* | 0.45,  >0.05 | *F_(1, 392),_*  *p* | **34.22,**  **<0.0001** |
|  | **Theta eyes open** | *F_(6, 392),_ p* | 0.47,  >0.05 | *F_(5, 392),_*  *p* | 1.22,  >0.05 | *F_(30, 392),_ p* | 0.40,  >0.05 | *F_(1, 392),_*  *p* | 0.02,  >0.05 |
|  | **Theta eyes closed** | *F_(6, 392),_ p* | 1.46,  >0.05 | *F_(5, 392),_*  *p* | 0.93,  >0.05 | *F_(30, 392),_ p* | 0.91,  >0.05 | *F_(1, 392),_*  *p* | 0.31,  >0.05 |
| **Occipital** | **Alpha eyes open** | *F_(6, 392),_ p* | 0.57,  >0.05 | *F_(5, 392),_*  *p* | 1.38,  >0.05 | *F_(30, 392),_ p* | 0.94,  >0.05 | *F_(1, 392),_*  *p* | **7.43,**  **<0.01** |
|  | **Alpha eyes closed** | *F_(6, 392),_ p* | 0.54,  >0.05 | *F_(5, 392),_*  *p* | 0.71,  >0.05 | *F_(30, 392),_ p* | 1.00,  >0.05 | *F_(1, 392),_*  *p* | **11.27,**  **<0.001** |
|  | **Theta eyes open** | *F_(6, 392),_ p* | 0.36,  >0.05 | *F_(5, 392),_*  *p* | 0.23,  >0.05 | *F_(30, 392),_ p* | 1.02,  >0.05 | *F_(1, 392),_*  *p* | 0.26,  >0.05 |
|  | **Theta eyes closed** | *F_(6, 392),_ p* | 0.92,  >0.05 | *F_(5, 392),_*  *p* | 1.13,  >0.05 | *F_(30, 392),_ p* | 1.21,  >0.05 | *F_(1, 392),_*  *p* | 0.71,  >0.05 |

**
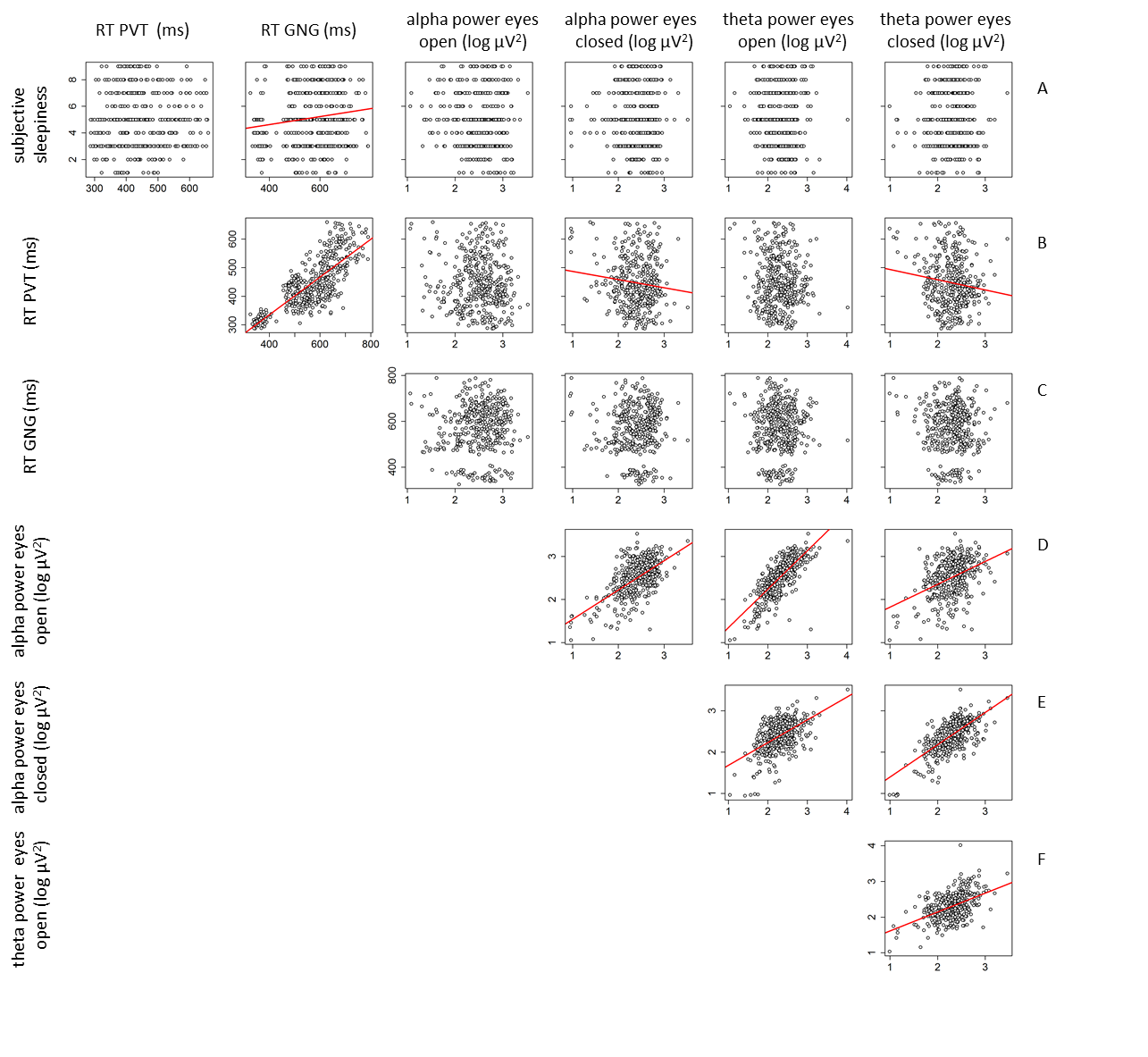
Figure S11:** **Correlation matrix of parameters of alertness.** Every dot represents one data point. Depicted are the correlation between subjective alertness and reaction time on the PVT, Go-NoGo and parameters of EEG (A), reaction time on the PVT, GNG, and EEG parameters (panel B), GNG reaction time and EEG parameters (C), and of EEG measures (D-F). Significant correlations (p<0.05) are indicated by a red line.
